# Epithelial redox stress programs macrophage immunometabolism through a ZNF24-MIF–NF–κB pathway in chronic nonbacterial prostatitis

**DOI:** 10.1016/j.redox.2026.104042

**Published:** 2026-01-20

**Authors:** Fei Zhang, Andong Zhang, Tong Meng, Xianhong Liu, Cheng Yang, Chaozhao Liang, Meng Zhang

**Affiliations:** Department of Urology, The First Affiliated Hospital of Anhui Medical University, Institute of Urology, and Anhui Province Key Laboratory of Genitourinary Diseases, Anhui Medical University, 218 Jixi Road, Shushan District, Hefei, Anhui, 230022, People's Republic of China

**Keywords:** Chronic nonbacterial prostatitis, MIF, CD74, PKM2, M1 polarization

## Abstract

Chronic nonbacterial prostatitis (CNP) is a prevalent and refractory urogenital disorder whose immunopathogenic mechanisms remain incompletely understood. Given that redox imbalance is increasingly recognized as a critical driver of chronic inflammation, this study systematically investigated the role of epithelial redox stress in immune regulation during CNP and its underlying molecular mechanisms. By integrating plasma cytokine profiling, bulk and single-cell transcriptomic analyses, and experimental autoimmune prostatitis (EAP) models, we identified epithelial-derived macrophage migration inhibitory factor (MIF) as a central mediator driving chronic prostatic inflammation. Mechanistically, inflammatory injury induced excessive accumulation of reactive oxygen species (ROS) in epithelial cells, which in turn activated the redox-responsive transcription factor ZNF24 to bind the MIF promoter and promote its transcription. Epithelial cell-derived MIF acted in a paracrine manner on CD74-expressing macrophages. Engagement of CD74 by MIF stabilized PKM2 expression, enhanced macrophage glycolytic reprogramming, promoted PKM2 nuclear translocation, and activated NF-κB-dependent transcriptional programs, thereby driving M1 macrophage polarization and proinflammatory cytokine production. Pharmacological interventions targeting distinct key nodes of this signaling pathway-including inhibition of MIF (ISO-1), blockade of CD74 (neutralizing antibodies), stabilization of PKM2 tetramers (DASA-58), and suppression of NF-κB (JSH-23)-significantly attenuated prostatic inflammation, restored mitochondrial homeostasis, and alleviated pelvic pain *in vitro* or *in vivo*. Collectively, these findings define an epithelial ROS-ZNF24-MIF-macrophage CD74-PKM2-NF-κB signaling axis, through which coordinated enhancement of glycolytic reprogramming and inflammatory signaling promotes M1 macrophage polarization and drives the initiation and progression of CNP. Moreover, multiple redox-sensitive nodes within this pathway represent promising therapeutic targets for precision immunomodulation in CNP.

## 1 Introduction

Chronic nonbacterial prostatitis (CNP) represents a common urological condition affecting adult males that is characterized by ongoing pelvic pain and lower urinary tract-related symptoms in the absence of detectable bacterial infection [1,2]. CNP impacts up to 10 % of men globally and contributes notably to psychological burden and financial strain for both individuals and healthcare systems [[3], [4], [5], [6]]. Although decades of clinical observations and empirical treatments have provided symptomatic relief, the underlying pathogenesis remains elusive, and current therapies often yield unsatisfactory outcomes.

Recent studies have focused on chronic, unresolved inflammation as a central driver of CNP progression, with prostate tissues from patients and experimental autoimmune prostatitis (EAP) models consistently exhibiting immune cell infiltration, particularly that of macrophages [[7], [8], [9], [10], [11]]. These infiltrating macrophages undergo polarization into proinflammatory M1 cells, which secrete TNF-α, IL-1β and IL-6 to sustain tissue injury, or into anti-inflammatory M2 cells, which secrete TGF-β and IL-10, which promote resolution and repair [12]. A predominance of the M1 phenotype establishes a self-amplifying circuit of cytokine release, tissue damage and further macrophage recruitment, thereby perpetuating pelvic pain and urinary dysfunction [13]. Pharmacological blockade of M1 polarization in EAP mice markedly reduces prostatic inflammation, underscoring the therapeutic potential of targeting macrophage plasticity [10].

In chronically inflamed tissues, epithelial cells are not merely passive targets of immune-mediated injury but actively shape the inflammatory microenvironment through redox-regulated signaling. Reactive oxygen species (ROS) have emerged as critical initiators and amplifiers of chronic inflammation via their ability to modulate transcriptional programs, cytokine production, and intercellular communication [14,15]. Excessive epithelial ROS accumulation not only causes oxidative macromolecular damage but also reshapes the inflammatory niche by activating redox-sensitive signaling pathways [[16], [17], [18]]. However, whether epithelial redox stress directly controls macrophage-activating cytokines and thereby orchestrates immune activation in CNP remains largely unexplored.

Macrophage migration inhibitory factor (MIF) is a pleiotropic proinflammatory cytokine with a wide range of immunoregulatory functions and is broadly expressed across multiple cell types, including immune, epithelial, endothelial, and neuronal cells [19]. Its contribution to autoimmune pathology is well documented in several autoimmune disorders, including ulcerative colitis, systemic lupus erythematosus, and rheumatoid arthritis [20]; for example, in the MRL/lpr murine lupus model, increased MIF expression in renal and cutaneous lesions is correlated with disease progression, and administration the small-molecule MIF inhibitor ISO-1 markedly decreases leukocyte infiltration and inflammatory cytokine production [21]. In the prostate, epithelial cells not only provide structural integrity but also actively modulate local immune responses [22]. Indeed, MIF expression is substantially increased in the prostatic epithelium of individuals diagnosed with benign prostatic hyperplasia (BPH) compared with that of normal controls [23], suggesting a potential role in prostatic disease. However, the function of MIF in CNP remains undefined.

MIF mediates its biological actions mainly through interaction with its receptor CD74, a type II membrane glycoprotein initially identified as the invariant chain of MHC class II complexes [24], which is abundantly expressed on macrophages and B lymphocytes, and is also detectable on other hematopoietic cells, such as T lymphocytes [25,26]. Genetic ablation of CD74 ameliorates a range of autoimmune disorders, including chronic glomerulonephritis, experimental autoimmune encephalomyelitis and systemic lupus erythematosus [27,28]. Pyruvate kinase M2 (PKM2), which serves as a key regulator of macrophage metabolic activity and polarization, exists in low-activity monomeric/dimeric and high-activity tetrameric forms [29]; Hu et al. [30] recently demonstrated that PKM2 modulates glycolytic flux and M1 polarization in intestinal macrophages. Although the MIF‒CD74 axis has been implicated in various autoimmune diseases, its role in orchestrating M1 macrophage polarization in CNP has not been systematically explored.

In the present study, we combined patient-derived biospecimens, single-cell and spatial transcriptomics, and EAP mouse models to dissect the epithelial-macrophage signaling network in CNP. We demonstrate that epithelial-derived MIF functions as a key paracrine effector driving macrophage M1 polarization through the MIF-CD74-PKM2-NF-κB-glycolysis cascade. More importantly, we identify epithelial ROS accumulation as the primary upstream event that activates the redox-responsive transcription factor ZNF24 to directly induce MIF transcription. These findings establish a previously unrecognized epithelial ROS-ZNF24-MIF-CD74 signaling axis that mechanistically links redox stress to immunometabolism reprogramming and CNP.

## 2 Materials and methods

### 2.1 Participant recruitment and sample collection

Plasma specimens were collected from individuals exhibiting chronic prostatitis-like symptoms (CP-LS) at the Department of Urology in our institution. Each participant completed the National Institutes of Health Chronic Prostatitis Symptom Index (NIH–CPSI) questionnaire to assess the severity of their symptoms [10]. The inclusion standards included: (1) persistent pelvic or perineal pain lasting over three months, accompanied by an NIH–CPSI pain domain score of 4 or higher; (2) first-time clinic attendance without coexisting urological diseases, such as urinary tract infections, acute/chronic bacterial prostatitis, urinary calculi, genitourinary tuberculosis); (3) no antibiotic therapy within the preceding three months; (4) prostate-specific antigen (PSA) ≤ 4.0 ng/mL; (5) transrectal ultrasound showing prostate dimensions <2 × 3 × 4 cm or postvoid residual urine <50 mL; and (6) age ≤50 years. Forty patients diagnosed with CP-LS and thirteen age-matched healthy controls recruited from the hospital's routine health examination center were enrolled.

Prostate tissues were obtained from eighty individuals diagnosed with BPH during transurethral resection procedures. Patients with incidental prostate carcinoma, preoperative prostatic intraepithelial neoplasia, indwelling catheter requirement, or ongoing urinary tract infection were excluded from the study. All human samples mentioned above were obtained from participants who had signed written informed consent forms prior to specimen collection. The study protocol was conducted in accordance with the Declaration of Helsinki and approved by the Ethics Committee of the First Affiliated Hospital of Anhui Medical University (Approval Nos. PJ-2020-07-11 and PJ-2021-03-13). The criteria for evaluating the degree of prostatitis in patients with BPH are listed in Table S1.

### 2.2 Establishment of the EAP model and behavioral assessment

Five-week-old nonobese male diabetic/LtJ (NOD) mice (18 ± 2 g; Jiangsu Jicui Yaokang Biotechnology, JingNan, China) were housed under SPF conditions at the Anhui Medical University Animal Center. All procedures were reviewed and authorized by the Anhui Medical University Institutional Animal Care and Use Committee (Approval Ethics Number: LLSC20241750). To induce EAP, prostate tissue was harvested from Sprague‒Dawley rats, homogenized and centrifuged, and the resulting supernatant was emulsified 1:1 (v/v) with complete Freund's adjuvant for prostate antigen (PAG) preparation [10]. On Day 0 and Day 28, NOD mice received subcutaneous injections of 300 μg PAG (50 μg per site) at six sites (bilateral lower abdomen, tail base and hind paw pads); control animals were injected with an equivalent emulsion of complete Freund's adjuvant and sterile saline.

Following the second immunization, the mice were treated daily for 14 days with ISO-1 (3.5 mg/kg in 10 % dimethyl sulfoxide (DMSO)/90 % corn oil; Cat. No. HY-16692; MCE) [31] via intraperitoneal injection, whereas the controls received vehicle alone. In parallel, the mice received intravenous injections of anti-CD74 neutralizing antibodies (750 μg/kg in phosphate-buffered saline (PBS); Cat. No. 555317, BD Pharmingen) [32] three times per week for 14 days; the control mice were given isotype IgG at the same dose and schedule. All the animals were sacrificed on day 42.

On the day prior to sacrifice, mechanical allodynia was assessed in the region of the lower abdomen adjacent to the prostate via Von Frey filaments. A nociceptive response was considered positive when the animal exhibited one or more of the following reactions during filament stimulation: (1) contraction of the abdomen, indicating pain perception; (2) prompt licking or scratching at the stimulated area, indicating localized discomfort; or (3) jumping, indicating a strong aversive response. The percentage of positive responses was recorded to quantify the sensitivity to mechanical stimulation.

### 2.3 Hematoxylin‒eosin (H&E) staining

Prostate glands were excised and fixed in 4 % paraformaldehyde for at least 24 h (h). Subsequently, samples were processed through routine histological procedures, embedded in paraffin, and sectioned into 4-μm slices. After being dried at 65 °C for 2 h, the sections underwent deparaffinization, rehydration, and washing, followed by hematoxylin-eosin staining, dehydration, clearing, and mounting in neutral resin. As previously described [22], inflammation within the prostate of EAP mice was semi-quantitatively evaluated on a four-grade scale (0–3), with the specific scoring criteria summarized in Table S2.

### 2.4 Cell culture and treatment of RWPE-1 and iBMDM cells

The iBMDM cell line (a murine immortalized macrophage line) was kindly provided by academician Feng Shao, and the human prostate epithelial cell line RWPE-1 (Cat. No. CL-0200, Procell) was obtained from Procell. iBMDM cells were cultured in high-glucose DMEM containing 10 % FBS and 1 % penicillin-streptomycin and passaged as needed. For pharmacological treatments, iBMDMs were pretreated with DASA-58 (50 μM, Cat. No. HY-19330, MCE) [33] or JSH-23 (10 μM, Cat. No. HY-13982, MCE) [34] for 1 h, followed by stimulation with recombinant MIF (100 ng/mL, Cat. No. CM059-20 MP, CHAMOT) for 24 h. To knock down CD74 expression in iBMDMs, the cells were transfected with CD74-specific siRNAs via Lipofectamine 3000 (Thermo Fisher Scientific) and incubated for 24 h prior to subsequent assays. Similarly, ZNF24 expression in RWPE-1 cells was silenced using ZNF24-targeting siRNAs following the same transfection procedure. The siRNA sequences employed are summarized in Table S3. The most efficient CD74-silencing siRNA was selected for all subsequent experiments. RWPE-1 cells were cultured and passaged in KM medium (Cat. No. 2101, ScienCell) supplemented with keratinocyte growth supplement according to the supplier's instructions. To establish an *in vitro* model of chronic prostatitis, lipopolysaccharide (LPS, 2.5 μg/mL; Cat. No. HY-D1056, MCE) was added to the culture medium of RWPE-1 cells for 6 h [22]. To ensure consistent conditions, all experiments used cells with fewer than ten passages and active proliferation. The antibodies and related reagents are listed with full specifications in Table S4 and S5**.**

### 2.5 Immunohistochemistry (IHC)

Paraffin-embedded prostate tissues underwent deparaffinization and rehydration following the procedure used for H&E staining. Antigen retrieval was performed in citrate buffer (pH 6.0) at 95 °C for 10 min, followed by PBS rinses. Endogenous peroxidase was quenched with 3 % hydrogen peroxide for 25 min at room temperature in darkness. After washing, the sections were incubated with 5 % BSA for 30 min to minimize nonspecific antibody binding.

Primary antibodies against MIF, CD74, and p-p65 were diluted in antibody buffer, applied to tissue sections, and incubated overnight at 4 °C. The slides were subsequently washed three times in PBS and treated with HRP-linked secondary antibodies for 1 h at room temperature in the dark. Following PBS washes, immunoreactivity was visualized via DAB substrate; the reaction was monitored under a microscope and terminated by rinsing in distilled water once optimal staining was achieved. Nuclei were counterstained with hematoxylin, and the sections were dehydrated through graded alcohols, cleared in xylene, and mounted with neutral resin. The stained slides were examined and imaged via a light microscope.

### 2.6 Immunofluorescence (IF)

Prostate tissue sections were prepared as described for IHC. For cell-based staining, culture slides were fixed with 4 % paraformaldehyde at ambient temperature for 15–30 min. To stain for intracellular antigens, selected tissue sections and cell samples were treated with 0.1 % Triton X-100 for 10 min for 10 min and PBS rinses. The slides were appropriately blocked and then incubated overnight at 4 °C with primary antibodies against MIF, epithelial cell adhesion molecule (EPCAM), CD74, CD86, CD45, CD68, iNOS, 8-OHDG, p65, and PKM2. After PBS washes, samples were incubated with fluorophore-conjugated secondary antibodies for 2 h at room temperature in darkness. DAPI was used for nuclear staining, and fluorescence images were obtained microscopically.

### 2.7 Western blot

Total proteins were isolated from both tissue and cellular samples using RIPA lysis buffer containing protease and phosphatase inhibitors. The protein concentrations were quantified using a BCA protein assay kit. Equivalent protein quantities were loaded onto 12.5 % SDS-PAGE gels for electrophoretic separation, followed by transfer onto nitrocellulose (NC) membranes, which were then blocked with 5 % nonfat milk in TBST for 1 h at room temperature. The membranes were incubated overnight at 4 °C with primary antibodies against MIF, CD74, UB, iNOS, CD86, p65, p-p65, PKM2, *p*-PKM2, ZNF24, β-actin, and Lamin B. After TBST washes, the membranes were incubated with HRP- linked secondary antibodies for 1 h. Protein signals were visualized with enhanced chemiluminescence (ECL) reagent and captured using Tanon detection system (Tanon, Shanghai, China).

### 2.8 RNA extraction and quantitative real-time polymerase chain reaction (qRT‒PCR)

Total RNA from tissues and cells was extracted using TRIzol reagent. RNA concentration and purity were measured spectrophotometrically. First-strand cDNA was generated from 1 μg of total RNA via the PrimeScriptTM RT Reagent Kit (Cat. No. RR047A; Takara). qRT-PCR was subsequently performed using the TB Green® Premix Ex TaqTM Kit (Cat. No. RR820A; Takara). The primer sequences used in this study are provided in Table S3. Relative mRNA expression levels were calculated via the 2^−ΔΔCt^ method and normalized to that of *ACTB*.

### 2.9 Enzyme-linked immunosorbent assay (ELISA)

The concentrations of IL-6 (Cat. No. E-EL-M0044, Elabscience), IL-1β (Cat. No. E-EL-M0037, Elabscience), and TNF-α (Cat. No. E-EL-M3063, Elabscience) were determined in NOD mouse serum and conditioned media from treated iBMDMs via commercial ELISA kits according to the manufacturer's protocols. MIF levels were quantified in NOD mouse serum (Cat. No. E-EL-M0771, Elabscience) and in human serum and RWPE-1 cell supernatant (Cat. No. E-EL-H6170, Elabscience).

### 2.10 Reactive oxygen species (ROS) assay

Intracellular ROS levels were assessed using a ROS detection kit (Cat. No. S0033S, Beyotime). RWPE-1 cells were rinsed with PBS and then incubated in fresh medium containing 10 μM DCFH-DA for 20 min. Fluorescence intensity was captured by fluorescence microscopy, and the mean fluorescence intensity (MFI) was quantified using ImageJ software (National Institutes of Health, Bethesda, MD, USA).

### 2.11 Flow cytometry

Single-cell suspensions were obtained from mouse spleens as previously reported [10]. The treated macrophages were rinsed twice with PBS and incubated at 4 °C for 1 h with the following fluorochrome-conjugated antibodies: anti-CD86-PE (Cat. No. 105008, BioLegend), anti-F4/80-FITC (Cat. No. 123108, BioLegend) and anti-CD11b-APC (Cat. No. 101212, BioLegend), and analyzed using a CytoFLEX flow cytometer (Beckman Coulter, USA). LPS (100 ng/ml)-stimulated macrophages were used as a positive control to verify CD86 antibody specificity. The data were processed via the manufacturer's software.

### 2.12 Co-culture

Mouse primary prostate epithelial cells were obtained from Procell (Cat. No. CP-M064, Procell). The cells were stimulated with LPS (2.5 μg/ml) for 6 h, after which the culture medium was then discarded, and cells were detached with 0.25 % trypsin-EDTA, washed three times with PBS, and seeded into the upper chamber of a six-well co-culture plate (Cat. No. 3412, Corning). iBMDMs were seeded in the lower wells of the same plate. Both cell types were maintained in DMEM/F-12 (Cat. No. 11330032, Gibco) supplemented with 10 % FBS and 1 % penicillin‒streptomycin for 24 h. Following co-culture, iBMDMs were harvested for following assays.

### 2.13 Chromatin immunoprecipitation (ChIP) assay

ChIP assay was performed using the SimpleChIP® Enzymatic Chromatin IP Kit (Cat. No. #9003, Cell Signaling Technology) following the manufacturer's instructions. The sequences for Primer (containing the ZNF24 binding site) were as follows:5′-CAGAGACCAAGGACAGGACCTCC-3' (forward) and 5′-TGAGGAGCTGAAGTTGCCCAG-3' (reverse). An anti-ZNF24 antibody (Cat. No. 11219-1-AP, Proteintech) was used for ChIP.

### 2.14 RNA sequencing

Total RNA was extracted from prostate tissues with TRIzol reagent, and RNA quality was evaluated using a NanoDrop 2000 spectrophotometer. Libraries were prepared with the VAHTS Universal V6 RNA-seq Kit (Vazyme) and sequenced on an Illumina NovaSeq 6000 platform (2 × 150 bp). FASTQ data were filtered and trimmed with fastp [35], aligned to the mouse reference genome (GRCm38) using HISAT2 [36]. Transcript abundance was calculated as fragments per kilobase of transcript per million mapped reads (FPKM) [37]. Read counts were generated by HTSeq-count [38] for downstream differential expression analysis.

### 2.15 Transmission electron microscopy (TEM)

iBMDM cells were fixed with 2.5 % glutaraldehyde in 0.1 M phosphate buffer (pH 7.4) at 4 °C for 2 h and postfixed with 1 % osmium tetroxide for 1 h. After ethanol dehydration and epoxy resin embedding, ultrathin sections (60–80 nm) were stained with uranyl acetate and lead citrate, and observed using a transmission electron microscope (Hitachi, Tokyo, Japan) at 80–120 kV to assess mitochondrial morphology and crista structure.

### 2.16 Mitochondrial membrane potential

The mitochondrial membrane potential (Δψm) was determined using the JC-1 assay kit (Cat. No. C2006, Beyotime) following the manufacturer's instructions. Briefly, Cells were incubated with JC-1 working solution at 37 °C for 20 min, washed with buffer, and observed under fluorescence microscopy (Olympus IX-73, Tokyo, Japan). JC-1 aggregates (high Δψm) emitting red fluorescence and monomers (low Δψm) emitting green fluorescence.

### 2.17 Lactate and glucose consumption assay

Treated iBMDMs were seeded in 6-well plates and maintained under the indicated conditions. The concentrations of lactate and glucose consumption of iBMDMs were measured via a lactate detection kit (Cat. No. A019-2-1; Nanjing Jiancheng, China) and a glucose consumption detection kit (Cat. No. F006-1-1; Nanjing Jiancheng, China) following the protocols provided by the manufacturer.

### 2.18 Coimmunoprecipitation, Coomassie brilliant blue staining and mass spectrometry

Interactions between CD74 and PKM2 were assessed via coimmunoprecipitation (co-IP) via an immunoprecipitation kit containing protein A/G magnetic beads (Cat. No. 88804; Thermo Fisher Scientific). The cells were lysed in 300 μL of IP lysis buffer, and the resulting lysates were clarified by centrifugation. The supernatants were incubated for 1 h with anti-IgG, anti-CD74 or anti-PKM2 antibodies at room temperature, followed by binding to pre-washed Protein A/G magnetic beads under gentle rotation for another 1 h. The beads were washed twice with IP wash buffer, and the bound proteins were eluted in elution buffer. The eluates were mixed with SDS loading buffer and boiled for 10 min.

Ten microliters of each immunoprecipitated sample was resolved via 12.5 % SDS‒PAGE at 80 V. The protein gels were visualized using Coomassie Brilliant Blue staining (Cat. No. 20279; Thermo Fisher Scientific). Distinct protein bands were excised and subjected to in-gel digestion and desalting. Peptides were dissolved in nano-HPLC buffer, separated on an Easy-nLC 1200 system (Thermo Fisher Scientific, Waltham, MA, USA), and then analyzed on a Thermo Scientific Lumos high-resolution mass spectrometer for protein identification.

### 2.19 Nuclear and cytosolic fractionation

Cytoplasmic and cellular nuclear fractions were isolated via a Nuclear Extract Kit (Cat. No. P0027, Beyotime) following the manufacturer's instructions.

### 2.20 Seahorse XF assays

Macrophages from different treatment groups were seeded at a density of 1.2 × 10^4^ cells per well into XFe96 plates. After attachment at room temperature, cells were treated with MIF (100 ng/mL) for 24 h. Meanwhile, the calibration solution and the probe plates containing sterile water were kept overnight in a 37 °C CO_2_-free incubator set. The next day, the sterile water in the probe plates was substituted with 200 μL of fresh calibration solution and incubated for an additional 60 min. The cells were subsequently washed with the prepared glycolysis stress test medium (2 mM glutamine) and the mitochondrial stress test medium (1 mM pyruvate, 2 mM glutamine, and 10 mM glucose), and the cell culture plates were placed in a 37 °C CO2-free incubator for 60 min. In the glycolysis stress test, the drugs used were glucose (10 mM), oligomycin (1.0 μM), and 2-DG (50 mM), whereas in the mitochondrial stress test, the drugs used were oligomycin (1.5 μM), FCCP (2 μM), and rotenone/antimycin A (0.5 μM). These drugs were loaded into different drug wells of the probe plates. The probe plates were subsequently calibrated on the Seahorse XF Pro analyzer. After calibration, the probe plates were replaced with cell culture plates, and the data were analyzed via Wave software.

### 2.21 Single-cell RNA sequencing

Prostate tissues from control and EAP mice were enzymatically and mechanically dissociated into single-cell suspensions following a standardized protocol. The cell suspensions were counted, adjusted to 1000 cells/μL, and loaded onto the MobiNova100 microfluidic platform (MobiCube) for droplet encapsulation, cell barcoding, reverse transcription, and cDNA preamplification via the High-Throughput Single-Cell 3′ Transcriptome Kit V2.1 (PN–S050200301) according to the manufacturer's instructions.

The full-length cDNA was enzymatically fragmented, end-repaired, A-tailed, adapter-ligated, and PCR amplification to construct sequencing libraries. Libraries were sequenced, and raw reads were processed with MobiVision QC software (v3.2; MobiCube) for alignment to the mouse reference genome (GRCm39), barcode and UMI identification, and low-quality read removal.

Downstream analysis was conducted in R (v4.4.0) via the Seurat package (v4.4.0). Low-quality cells were excluded on the basis of standard QC metrics, including gene counts, mitochondrial gene percentage, and ribosomal and hemoglobin gene content, to remove stressed or contaminant populations. Doublets were identified and excluded via the SCP algorithm [39]. The expression data were normalized with NormalizeData (LogNormalize, scale.factor = 1e4). Genes showing high variability across cells were identified through the FindVariableFeatures function. Scaled data were subjected to principal component analysis (RunPCA), and potential batch-dependent variations were adjusted via Harmony (RunHarmony, covariate = “orig.ident”).

Batch-corrected principal components were visualized via UMAP (RunUMAP on the top 15 PCs). Nearest neighbor graphs were constructed (FindNeighbor), and clustering was performed at multiple resolutions (FindClusters). Cluster relationships across resolutions were evaluated using clustree, and cell populations were annotated on the basis of differentially expressed genes (FindAllMarkers) in combination with canonical marker genes curated from the literature [[40], [41], [42]]. Cell type annotation for BPH tissues was performed according to our recent study [10]. Functional metabolic states were evaluated with ScMetabolism, with a focus on inflammatory macrophage subsets [43]. Intercellular communication networks were inferred via CellChat in human BPH tissues, and cell2location was applied for spatial deconvolution of cell subpopulations [[44], [45], [46]]. Commot was further used to visualize directional ligand‒receptor signaling, including the MIF‒CD74 axis [47]. Single-cell M1/M2 polarization scores and TNFA-via–NF–κB pathway activity were quantified using AUCell and the AddModuleScore function, respectively. Monocle3 was employed to reconstruct macrophage differentiation trajectories and dynamic gene expression changes [48].

### 2.22 Statistical analysis

All data are presented as the mean ± standard deviation (SD) or *M* (*Q1*, and *Q3*). Statistical comparisons between two groups were performed via an unpaired two-tailed Student's *t*-test, or comparisons among multiple groups were performed via one-way ANOVA followed by Bonferroni post hoc correction. Analyses were conducted in GraphPad Prism v10.6.0, and *P* < 0.05 was considered statistically significant.

## 3 Results

### 3.1 Epithelial-derived MIF is upregulated in CNP and is correlated with inflammation severity

**Fig. 1:**
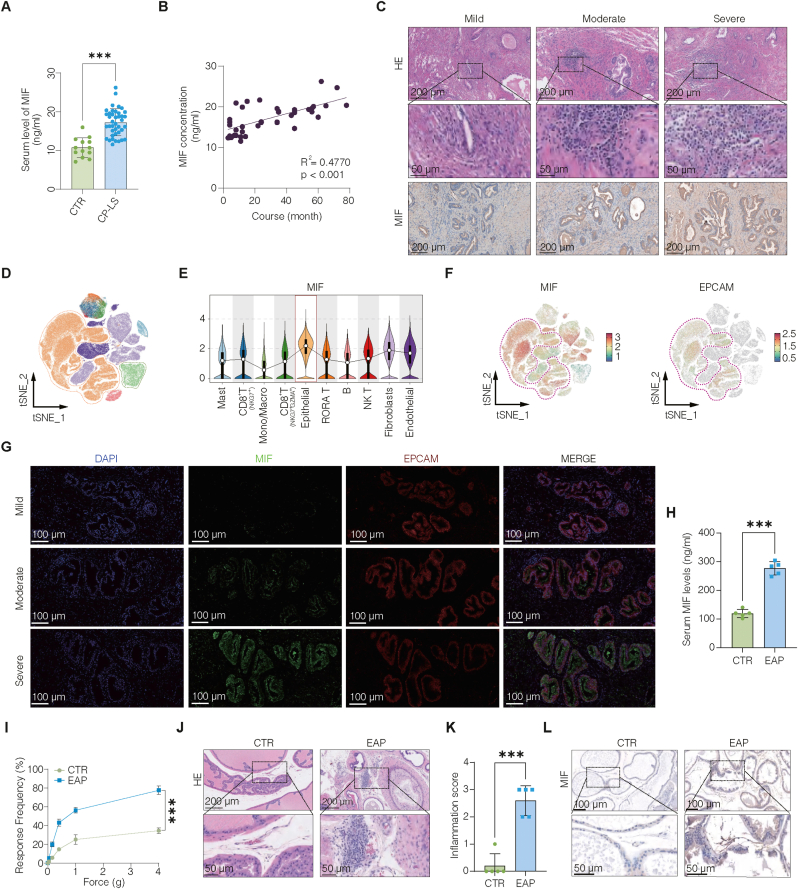
Epithelial-derived MIF is upregulated in CNP and is correlated with inflammation severity. (A) ELISA quantification of MIF protein levels in plasma samples from CP-LS patients (n = 40) and healthy controls (CTR, n = 13). (B) Correlation between MIF concentration and disease duration in CP-LS patients (Spearman's r = 0.4770, p < 0.001). (C) Representative H&E staining and MIF IHC of prostate tissues from BPH patients with mild, moderate, and severe inflammation, showing increasing MIF expression with inflammatory severity. (D) t-SNE plot of single-cell transcriptomic data from normal and BPH human prostate tissues, showing clustering of major cell types. (E) Violin plots of MIF expression across cell types, showing enrichment in epithelial cells. (F) t-SNE feature plots showing overlapping expression of MIF and the epithelial marker EPCAM, with enrichment in epithelial cells. (G) Immunofluorescence staining of BPH tissues showing co-localization of MIF (green) and EPCAM (red) with increasing inflammatory severity. (H) ELISA quantification of MIF levels in serum samples from EAP and CTR mice (n = 5/group). (I) Behavioral assessment of pelvic pain by von Frey testing in EAP versus CTR mice, showing increased mechanical allodynia in EAP. (J) Representative H&E staining of mouse prostate tissues showing inflammatory infiltrates in EAP. (K) Quantification of prostate inflammation scores in CTR and EAP mice (n = 5/group). (L) IHC staining of mouse prostate tissues showing elevated MIF expression in EAP. Data are presented as the mean ± SD. ns, not significant; ∗p < 0.05; ∗∗p < 0.01; ∗∗∗p < 0.001. **Abbreviations:** CP-LS, chronic prostatitis-like symptoms; CTR, control; EAP, experimental autoimmune prostatitis; t-SNE, t-distributed stochastic neighbor embedding.

To systematically identify cytokines associated with CNP, we performed a comprehensive profiling of 48 plasma cytokines in patients with CP-LS and age-matched healthy controls [10], which revealed broad inflammatory alterations. Among these factors, MIF showed a clear elevation in the initial screening and, given its known capacity to be actively produced by epithelial cells under stress conditions rather than merely reflecting downstream immune activation, was prioritized for further validation and mechanistic investigation. Subsequent targeted analyses demonstrated that MIF exhibited a stable and reproducible upregulation across independent patient samples (Fig. 1A), with expression levels showing a positive correlation with earlier symptom onset (r = 0.4770, p < 0.001; Fig. 1B–Table S6), implicating MIF in disease initiation. To delineate its cellular source and spatial localization, we conducted immunohistochemistry on BPH tissues, which revealed that MIF expression intensity was positively associated with inflammatory severity (Fig. 1C, and Fig. S1A). Analysis of public single-cell RNA-sequencing datasets further revealed that MIF expression was specifically enriched in epithelial cells (Fig. 1D–F, and Fig. S1B), a finding independently corroborated by immunofluorescence colocalization analysis (Fig. 1G, and Fig. S1C).

To assess the *in vivo* relevance of MIF, we examined its expression in the EAP mouse model. ELISA, immunohistochemistry, and immunofluorescence staining consistently revealed marked upregulation of MIF in inflamed prostatic tissues compared with healthy control tissues (Fig. 1H–L, and Fig. S1D–F). These findings, in conjunction with human data, reveal that epithelial cells constitute the major source of MIF under inflammatory conditions and imply that MIF may be involved in the pathogenesis of chronic prostatitis.

### 3.2 MIF‒CD74 signaling coordinates epithelial‒macrophage crosstalk in inflamed prostate tissue

**Fig. 2:**
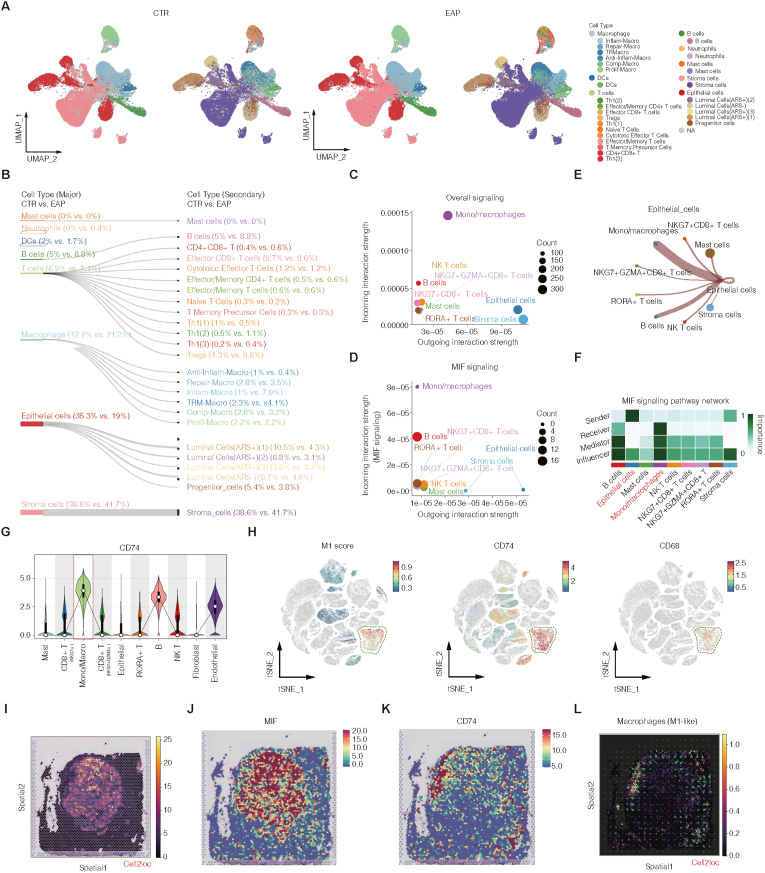
MIF-CD74 signaling coordinates epithelial-macrophage crosstalk in inflamed prostate tissue. (A) UMAP representation of single-cell RNA sequencing data from control (CTR) and EAP mouse prostates. (B) Sankey diagrams illustrating proportional changes in major and secondary cell populations, showing increased B cells, Th1(2) cells, and inflammatory macrophages (Inflam-Macro) in EAP. (C) CellChat analysis of human BPH single-cell transcriptomic data identifying monocytes/macrophages as the dominant signal-receiving population. (D) Within the MIF signaling pathway, epithelial cells act as the primary signal senders, whereas monocytes/macrophages represent the predominant recipients. (E) CellChat role analysis highlighting epithelial cells as major “Senders” and monocytes/macrophages as key “Receivers,” “Mediators,” and “Influencers” within the MIF network. (F) Heatmap summarizing the relative contribution of each cell type to the MIF signaling network based on role analysis. (G) Violin plots showing preferential expression of CD74 in monocytes/macrophages. (H) t-SNE feature plots of M1 module score, CD74, and CD68 expression, indicating enrichment of CD74 in M1-like macrophage subsets. (I–L) Spatial transcriptomic analysis using Cell2location revealing spatial co-localization of MIF-expressing epithelial cells (I–J) and CD74-expressing macrophages (K) within regions enriched for M1-like macrophages (L). **Abbreviations:** Abbreviations: CTR, control; EAP, experimental autoimmune prostatitis; UMAP, uniform manifold approximation and projection; Cell2location, spatial cell-type deconvolution tool; t-SNE, t-distributed stochastic neighbor embedding.

To characterize alterations in the immune microenvironment and identify the most prominently affected immune cell subsets in chronic prostatitis, we performed single-cell RNA sequencing on prostate tissues from EAP (n = 4) and control (n = 2) mice. The quality control metrics of the samples were assessed, and the quality control results are presented in Fig. S2. The results of the cell type annotation and functional characterization are shown in Fig. S3A–C. This analysis revealed increased infiltration of multiple immune subsets, notably B cells, Th1(2) cells, and inflammatory macrophages (Inflam-Macro), with the latter exhibiting the most prominent expansion (Fig. 2A–B). This enrichment suggests a pivotal role for inflammatory macrophages in the initiation and progression of chronic prostatic inflammation.

To uncover the intercellular signaling events underlying this response, we applied CellChat analysis to human single-cell transcriptomic data. Epithelial cells emerged as one of the predominant signal-sending population, whereas monocytes/macrophages were identified as the primary signal receivers across the global interaction network (Fig. 2C). Specifically, within the MIF pathway, epithelial cells displayed the highest outgoing communication probability, and monocytes/macrophages and B cells were the principal receiving compartments (Fig. 2D, and Fig. S3D). Notably, the epithelial-to-macrophage signaling channel ranked among the most robust interaction axes inferred (Fig. S3E). Functional role analysis within the MIF signaling network further revealed epithelial cells as dominant senders and monocytes/macrophages as not only the main receivers but also key mediators and influencers (Fig. 2E–F), indicating a dual role in receiving and amplifying inflammatory cues. Investigation of receptor expression revealed that CD74, a high-affinity receptor for MIF, was selectively enriched on monocytes/macrophages (Fig. 2G). Moreover, module scoring demonstrated that *CD74* expression was tightly colocalized with macrophage clusters exhibiting an M1-polarized transcriptional phenotype (Fig. 2H), implicating this MIF‒CD74 axis in proinflammatory reprogramming.

To spatially resolve this signaling circuit, we utilized cell2location-based deconvolution of spatial transcriptomics data. Striking co-localization was observed between MIF-producing epithelial cells (Fig. 2I–J) and *CD74*-expressing macrophages (Fig. 2K), particularly within regions enriched for M1-like macrophage infiltration (Fig. 2L*,* Figure S3F and Figure S4). These data collectively reveal a spatially organized, epithelial-to-macrophage MIF‒CD74 signaling axis in chronic prostatitis, supporting a model in which epithelial stress drives localized immune activation through directed proinflammatory communication.

### 3.3 Inflammatory M1-like macrophages act as terminal effector cells driven by epithelial-derived MIF-CD74 signaling in chronic prostatitis

**Fig. 3:**
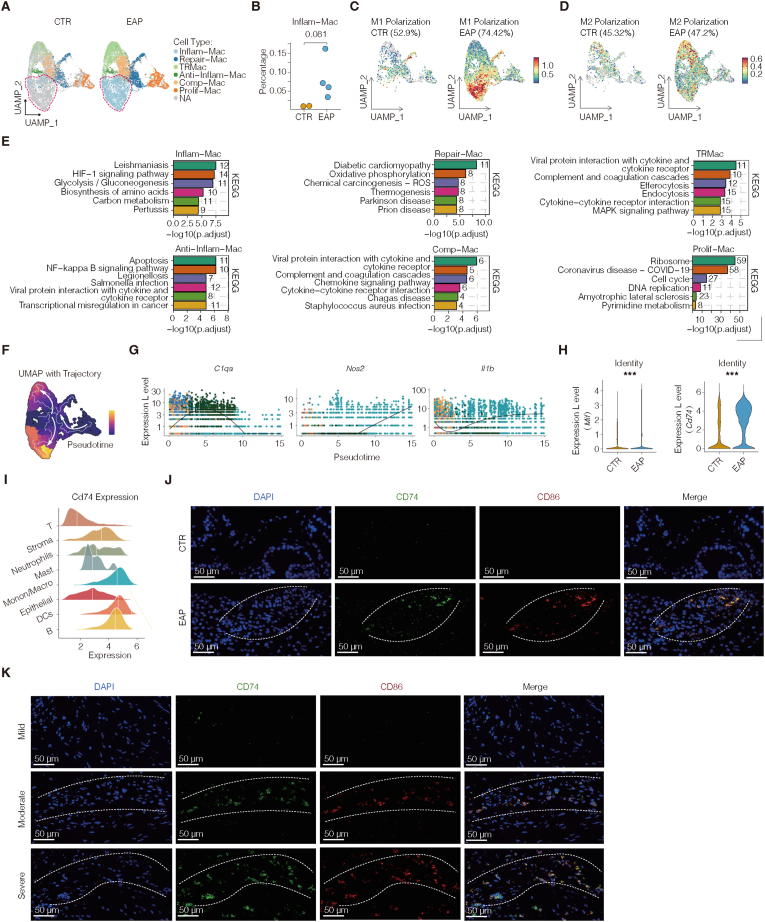
Inflammatory M1-like macrophages act as terminal effector cells driven by epithelial-derived MIF-CD74 signaling in chronic prostatitis. (A) UMAP plots of macrophage subtypes in CTR and EAP mouse prostates. (B) Frequencies of macrophage subpopulations, showing expansion of inflammatory macrophages (Inflam-Mac) in EAP. (C) AUCell-based scoring of M1-like polarization signatures across macrophage subsets. (D) AUCell-based scoring of M2-like polarization signatures across macrophage subsets. (E) KEGG pathway enrichment analysis of differentially expressed genes in macrophage subsets, indicating inflammatory and immunomodulatory programs. (F–G) Monocle pseudotime analysis revealing distinct trajectories, with *C1qa* expression declining and *Nos2*/*Il1b* increasing during transition from proliferating to inflammatory macrophages. (H) *Mif* and *Cd74* expression across CTR and EAP, showing upregulation in EAP. (I) Ridge plots of *Cd74* expression across immune cell types, highlighting macrophage enrichment. (J) Multiplex immunofluorescence of mouse prostates showing co-localization of CD74 (green) and CD86 (red) in EAP versus CTR; nuclei stained with DAPI (blue). Scale bars, 50 μm. (K) Multiplex immunofluorescence of human BPH tissues showing increased CD74^−^CD86 co-localization in regions with moderate/severe versus mild inflammation. Data are presented as the mean ± SD. ns, not significant; ∗p < 0.05; ∗∗p < 0.01; ∗∗∗p < 0.001. **Abbreviations:** CTR, control; EAP, experimental autoimmune prostatitis; UMAP, uniform manifold approximation and projection; AUCell, area under the curve for gene set activity; KEGG, Kyoto Encyclopedia of Genes and Genomes.

Given the prominent role of macrophages in chronic inflammation, we next characterized their distribution and abundance in EAPs and control prostates. UMAP visualization combined with boxplot analysis revealed distinct differences in macrophage distributions and proportions between the two groups (Fig. 3A–B, and Fig. S5A). To further classify macrophages on the basis of their polarization state, we applied the AUCell scoring method to quantify transcriptional programs associated with M1-like and M2-like phenotypes. Inflammatory macrophages derived from EAP prostates presented elevated M1 scores and robust expression of canonical proinflammatory genes, including *Nos2*, *Il1b*, *Tnf*, and *Cd86* (Fig. 3C–D, and Fig. S5B), indicating classical M1-like activation.

Functional enrichment analysis via the KEGG database revealed distinct signaling characteristics among macrophage subpopulations. Inflam-Macro genes were significantly enriched in pathways related to leishmaniasis, HIF-1 signaling, and glycolysis/gluconeogenesis, suggesting activation through hypoxia-induced metabolic reprogramming and microbial pattern recognition. Conversely, tissue-resident macrophages (TRMacs) were enriched predominantly for complement and coagulation cascades, cytokine‒receptor interactions, and efferocytosis pathways, reflecting regulatory and homeostatic roles (Fig. 3E). Trajectory analysis via Monocle pseudotime revealed that Inflam-Macro and TRMacs resided at separate terminal states along divergent differentiation trajectories (Fig. 3F). During the transition from proliferating macrophages to inflammatory subsets, the *C1qa* gene, which is linked to phagocytic tolerance, was progressively downregulated, whereas *Nos2* and *Il1b* were upregulated, indicating the acquisition of a mature proinflammatory phenotype (Fig. 3G).

Given the central role of the MIF‒CD74 axis in macrophage activation, we next examined the expression of *Mif* and its receptor *Cd74*. Both genes were significantly upregulated in EAP mice relative to controls (Fig. 3H). Ridge plot analysis and cell-type mapping confirmed that *Cd74* was predominantly expressed in monocyte/macrophage clusters (Fig. 3I). These transcriptomic findings were validated by multiplex immunofluorescence: in EAP mouse prostates, CD74 showed strong spatial co-localization with the M1 macrophage marker CD86, with markedly increased signal intensity relative to that in controls (Fig. 3J). Similarly, in human BPH tissues, the co-expression of CD74 and CD86 was more pronounced in areas with moderate to severe inflammation than in mildly inflamed regions (Fig. 3K). These findings identify M1-like inflammatory macrophages as terminal effector cells that expand in chronic prostatitis, with the MIF‒CD74 axis acting as a key epithelial‒macrophage signaling pathway sustaining immune activation.

### 3.4 Pharmacological inhibition of MIF by ISO-1 attenuates prostatitis severity and suppresses M1 macrophage polarization *in vivo*

**Fig. 4:**
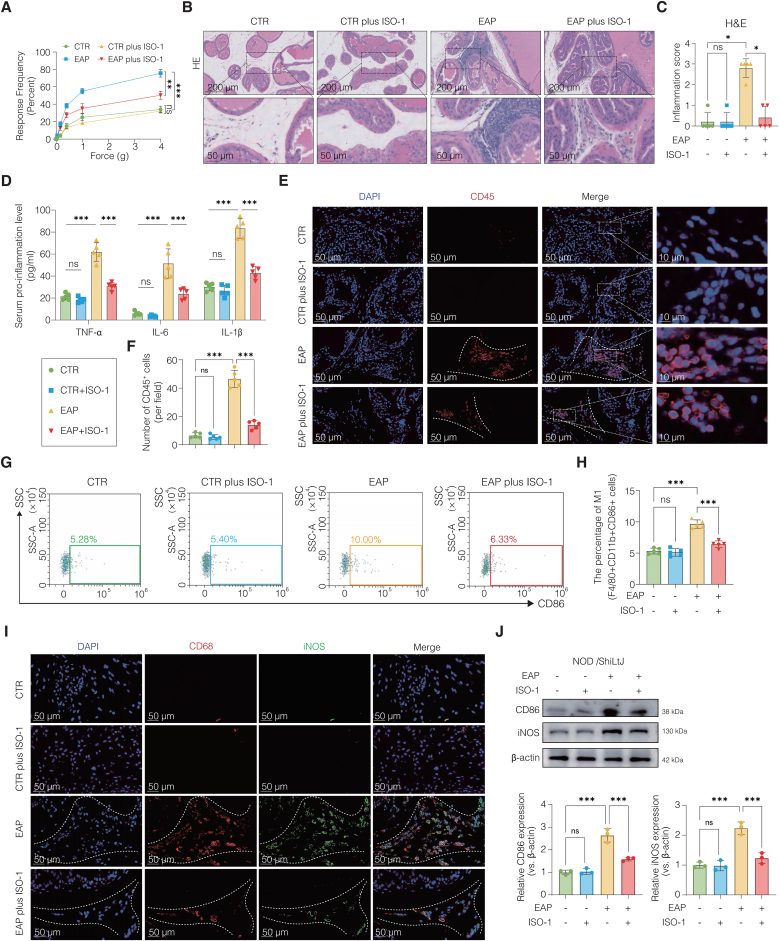
Pharmacological inhibition of MIF by ISO-1 attenuates prostatitis severity and suppresses M1 macrophage polarization *in vivo*. (A) Mechanical allodynia assessed by von Frey testing, showing reduced response frequency in ISO-1-treated mice. (B) Representative H&E staining of prostate sections showing decreased inflammatory infiltration after ISO-1 treatment. (C) Quantification of histopathological inflammation scores. (D) ELISA quantification of TNF-α, IL-6, and IL-1β in serum confirming cytokine downregulation. (E) Immunofluorescence staining for CD45 (red) indicating reduced immune cell infiltration. (F) Quantification of CD45^+^ immune cell infiltration in prostate tissues. (G–H) Flow cytometry of F4/80+CD11b + CD86^+^ macrophages showing reduced M1 subset after ISO-1. (I) Co-immunofluorescence for CD68 (red) and iNOS (green) showing reduced M1 macrophages in ISO-1-treated mice. (J) Western blot confirming downregulation of CD86 and iNOS in prostate tissues. Data are presented as the mean ± SD. ns, not significant; ∗p < 0.05; ∗∗p < 0.01; ∗∗∗p < 0.001. **Abbreviations:** CTR, control; EAP, experimental autoimmune prostatitis; qRT‒PCR, quantitative real-time PCR.

To elucidate the functional role of MIF in the pathogenesis of CNP and its influence on macrophage polarization, we administered the selective MIF inhibitor ISO-1 to EAP mice (Fig. S6A). ISO-1 treatment markedly alleviated prostatitis-related symptoms, as evidenced by a reduced response frequency and inflammatory cell infiltration (Fig. 4A–C). qRT‒PCR analysis revealed that the proinflammatory cytokines *Tnf*, *Il1b*, and *Il6* were significantly upregulated in the EAP group and were notably suppressed following ISO-1 treatment (Fig. S6B). These results were corroborated by ELISA, which confirmed reduced cytokine levels upon MIF inhibition (Fig. 4D).

Immunofluorescence staining revealed a marked reduction in CD45^+^ immune cell infiltration following ISO-1 administration, which was consistent with the H&E histological findings (Fig. 4E–F). Immunohistochemical staining further revealed a decrease in CD74 positive cells in treated animals (Fig. S6C). Western blot analysis supported these observations, indicating that CD74 protein levels, which were markedly elevated in the EAP group, were effectively suppressed by ISO-1 (Fig. S6D). Flow cytometry analysis demonstrated an increased proportion of M1-polarized macrophages in EAP mice, which was significantly reduced following ISO-1 treatment (Fig. 4G–H). Co-immunofluorescence staining for CD68 and iNOS further confirmed a reduction in M1 macrophage abundance in ISO-1-treated mice (Fig. 4I). Additionally, the protein levels of CD86 and iNOS, key markers of M1 polarization, were elevated in the EAP model and were substantially decreased following ISO-1 administration, as demonstrated by Western blot (Fig. 4J). Collectively, these results demonstrate that pharmacological inhibition of MIF by ISO-1 not only attenuates chronic prostatic inflammation but also suppresses M1 macrophage polarization, highlighting the MIF‒macrophage axis may represent a viable therapeutic target for chronic prostatitis.

### 3.5 Blockade of CD74 attenuates prostatitis severity and restrains M1 macrophage polarization *in vivo*

**Fig. 5:**
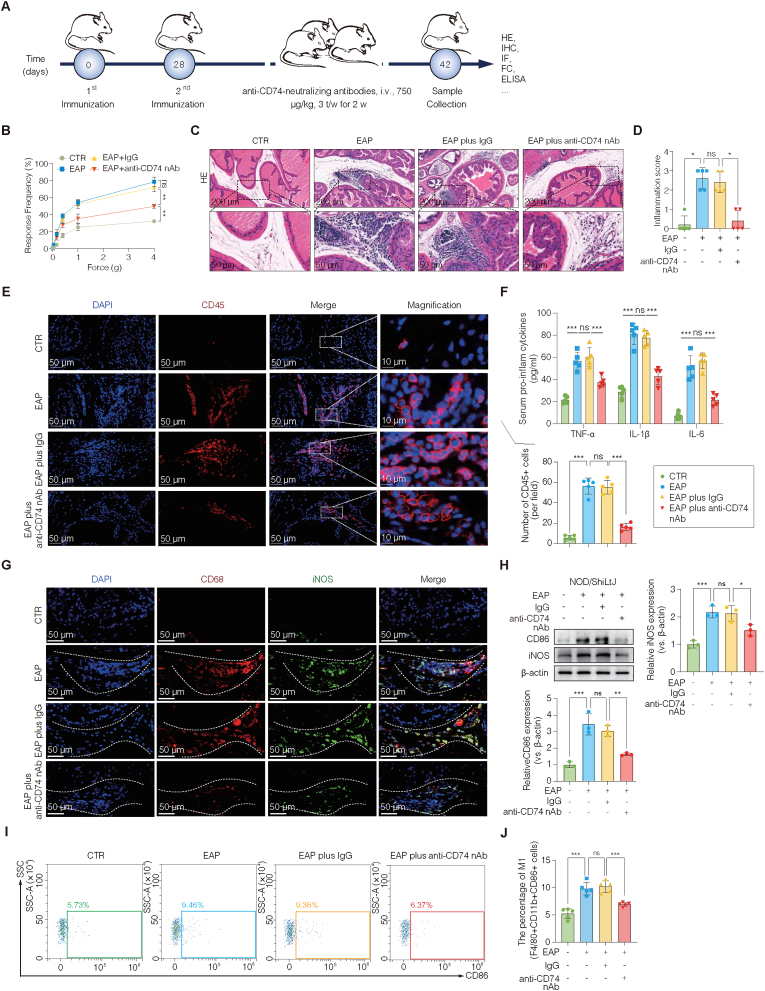
Blockade of CD74 attenuates prostatitis severity and restrains M1 macrophage polarization *in vivo*. (A) Experimental timeline for anti-CD74 neutralizing antibodies (CD74 nAb) administration in the CNP mouse model. The antibodies were delivered via tail vein injection (750 μg/kg, three times weekly) for two weeks before tissue collection on day 42. (B) Mechanical allodynia measured by von Frey testing showing reduced pain sensitivity in CD74 nAb-treated mice. (C) Representative H&E staining of prostate sections from the CTR, EAP, EAP + IgG, and EAP + CD74 nAb groups; the lower panels show magnified regions. (D) Quantification of histological inflammation scores. (E) Immunofluorescence of CD45 positive immune cells showing decreased leukocyte infiltration in the CD74 nAb group. (F) ELISA quantification of the serum TNF-α, IL-6, and IL-1β levels confirmed the downregulation. (G) Co-immunofluorescence for CD68 (macrophage marker) and iNOS (M1 marker) revealed reduced M1 macrophage infiltration after CD74 blockade. (H) Western blot analysis of CD86 and iNOS protein levels. (I–J) Flow cytometry of F4/80+CD11b + CD86^+^ macrophages, with reduced M1 polarization in the CD74 nAb group, as quantified in (J). The data are presented as the means ± SD. ns, not significant; ∗p < 0.05; ∗∗p < 0.01; ∗∗∗p < 0.001. **Abbreviations:** CD74 nAb, anti-CD74 neutralizing antibodies; CTR, control; EAP, experimental autoimmune prostatitis; qRT‒PCR, quantitative real-time PCR.

To further explore the role of the MIF receptor CD74 in CNP, we administered anti-CD74-neutralizing antibodies to EAP mice (Fig. 5A). Antibodies treatment led to a significant reduction in the clinical features of prostatitis, including decreased response frequency and diminished inflammatory cell infiltration, as shown by histological analysis (Fig. 5B–D). Immunofluorescence staining confirmed a notable decrease in CD45^+^ leukocyte infiltration within the prostate following CD74 blockade (Fig. 5E), which was consistent with the H&E findings. At the molecular level, qRT‒PCR revealed that the expression of the proinflammatory cytokines *Tnf*, *Il1b*, and *Il6* was substantially elevated in EAP mice and significantly reduced upon CD74 inhibition (Fig. S7). These transcriptional findings were further supported by ELISA, which revealed decreased cytokine secretion in the antibody-treated animals (Fig. 5F).

To assess macrophage polarization, we performed co-immunofluorescence staining for CD68 and iNOS, which revealed a marked reduction in M1-like macrophages following CD74 blockade (Fig. 5G). Western blot analysis verified the downregulation of the M1-associated markers CD86 and iNOS in treated animals, in contrast to their pronounced increase in the EAP group (Fig. 5H). Flow cytometry analysis further validated these findings, demonstrating a marked decrease in the proportion of M1-polarized macrophages upon anti-CD74 neutralizing antibodies treatment (Fig. 5I–J). Together, these results demonstrate that pharmacological blockade of CD74 effectively mitigates prostatic inflammation and restrains M1 macrophage polarization in the CNP model. These findings reinforce CD74 as a key mediator of MIF signaling and a viable therapeutic target in CNP.

### 3.6 Epithelial ROS-ZNF24 axis drives MIF transcription to promote CD74-dependent M1 macrophage polarization and proinflammatory activation

**Fig. 6:**
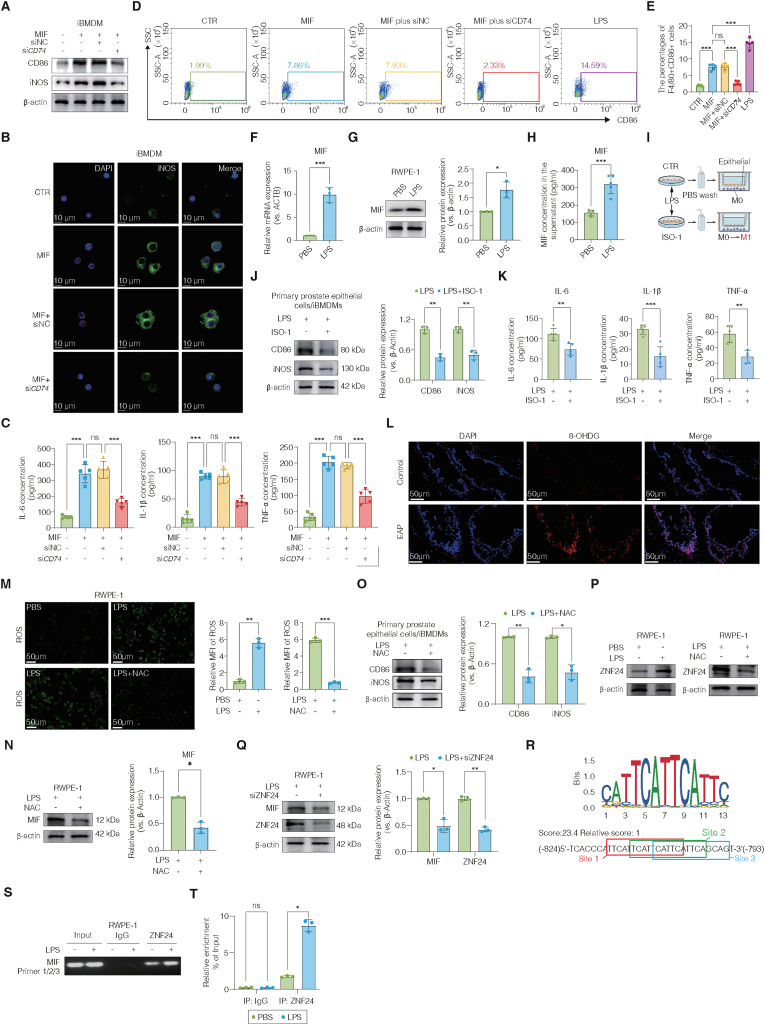
Epithelial ROS-ZNF24 axis drives MIF transcription and promotes CD74-dependent M1 macrophage polarization. (A–E) CD74 knockdown attenuates MIF-induced M1 macrophage polarization, as assessed by CD86 and iNOS expression (A–B), proinflammatory cytokine secretion (C), and flow cytometry analysis of F4/80^+^CD86^+^ macrophages (D–E). (F–H) LPS stimulation induces MIF mRNA expression (F), protein expression (G), and secretion (H) in RWPE-1 prostate epithelial cells. (I–K) Transwell co-culture system showing that LPS-stimulated prostate epithelial cells promote M1 polarization and cytokine secretion in iBMDMs, which is suppressed by the MIF inhibitor ISO-1. (L–M) Increased epithelial oxidative stress in EAP mice and LPS-stimulated RWPE-1 cells, indicated by 8-OHdG staining (L) and intracellular ROS levels (M), respectively; NAC effectively reduces ROS accumulation. (N–O) ROS scavenging with NAC suppresses epithelial MIF expression and attenuates M1 marker expression in co-cultured macrophages. (P–T) ZNF24 is induced by epithelial ROS and directly regulates MIF transcription, as shown by ZNF24 expression (P), ZNF24 knockdown (Q), predicted ZNF24 binding motifs in the MIF promoter (R), and ChIP assays demonstrating enhanced ZNF24 binding upon LPS stimulation (S–T). Data are presented as mean ± SD. ns, not significant; ∗p < 0.05; ∗∗p < 0.01; ∗∗∗p < 0.001. **Abbreviations:** iBMDMs, immortalized bone marrow-derived macrophages; RWPE-1, human prostate epithelial cell line; siCD74, small interfering RNA targeting CD74; LPS, lipopolysaccharide; NAC, N-acetylcysteine; ROS, reactive oxygen species.

Recombinant MIF induced the expression of CD86 and iNOS in iBMDMs in a dose-dependent manner, with the maximal effect observed at 100 ng/mL (Fig. S8A). Time-course analysis further demonstrated that stimulation with 100 ng/mL MIF for 24 h resulted in the strongest induction of these M1 markers (Fig. S8B). Silencing of CD74 via siRNA significantly attenuated MIF-induced upregulation of CD86 and iNOS, as confirmed by Western blot analysis (Fig. 6A, and Fig. S8C–D). Consistently, immunofluorescence staining showed that MIF-enhanced iNOS expression was markedly abolished upon CD74 knockdown (Fig. 6B, and Fig. S8E). Moreover, MIF treatment markedly increased the mRNA expression and secretion of the proinflammatory cytokines IL-1β, IL-6, and TNF-α, which were all significantly reversed by CD74 silencing, as determined by qRT-PCR and ELISA (Fig. 6C, and Fig. S8F). Flow cytometric analysis further confirmed that MIF-induced M1 polarization was substantially reduced following CD74 inhibition (Fig. 6D–E), indicating that MIF-driven macrophage M1 polarization is largely CD74-dependent.

To determine whether epithelial cells could serve as a source of MIF under inflammatory conditions, human prostate epithelial RWPE-1 cells were stimulated with LPS to mimic an inflammatory environment. Both mRNA and protein levels of MIF were significantly increased, as demonstrated by immunofluorescence, qRT-PCR, Western blot, and ELISA (Fig. 6F–H, Fig. S8G). To further investigate whether epithelial cell-derived MIF directly promotes macrophage M1 polarization, mouse primary prostate epithelial cells were subjected to a transwell co-culture system with macrophages. Macrophages co-cultured with LPS-stimulated epithelial cells exhibited enhanced expression of CD86 and iNOS, which was significantly reversed by the MIF inhibitor ISO-1 (Fig. 6I–J). Similarly, the elevated levels of IL-1β, IL-6, and TNF-α in macrophages were markedly suppressed after pharmacological blockade of MIF (Fig. 6K).

To determine whether oxidative stress serves as an upstream trigger for epithelial MIF induction, we first assessed the oxidative DNA damage marker 8-OHdG in prostate tissues from the EAP model and control mice. A marked elevation of 8-OHdG was observed in the EAP group (Fig. 6L), indicating pronounced epithelial oxidative stress under inflammatory conditions. In RWPE-1 cells, LPS stimulation markedly increased intracellular ROS levels, whereas pretreatment with the ROS scavenger N-acetylcysteine (NAC) significantly reduced ROS accumulation (Fig. 6M). Notably, pharmacological scavenging of ROS with NAC simultaneously downregulated LPS-induced MIF expression at both the mRNA and protein levels and markedly reduced its secretion into the culture supernatant, demonstrating that epithelial MIF induction is ROS-dependent (Fig. 6N–and Figure S8H). In parallel, NAC also significantly reduced the expression of the M1 markers CD86 and iNOS in the co-culture system of mouse prostate epithelial cells and iBMDM cells (Fig. 6O).

To explore the potential mechanism of MIF induction in CNP, the JASPAR 2022 database was used to predict transcription factor binding sites within its promoter region. High-scoring potential ZNF24 binding sites were identified in the *MIF* promoter (Supplementary Table S7, **Excel file**). Consistently, ReMap 2022 ChIP-seq datasets revealed significant enrichment of ZNF24 at the *MIF* promoter across multiple cell lines (Fig. S8I), supporting ZNF24 as a direct upstream regulator of MIF transcription. Furthermore, our results showed that LPS stimulation markedly increased ZNF24 expression in RWPE-1 cells, whereas this effect was largely abolished by the ROS scavenger NAC (Fig. 6P, and Fig. S8J), indicating that ZNF24 upregulation is mediated by LPS-induced ROS accumulation. To further verify the role of ZNF24 in ROS-mediated MIF regulation, three siRNAs targeting ZNF24 were designed, among which siRNA-3 showed the most efficient knockdown (Fig. S8K). Silencing of ZNF24 in RWPE-1 cells significantly suppressed the LPS-induced increases in *MIF* mRNA expression, protein levels, and extracellular secretion (Fig. 6Q, and Fig. S8L). Meanwhile, the predicted ZNF24 binding sites within the *MIF* promoter showed strong consistency with the canonical ZNF24 motif (Fig. 6R). ChIP assays further confirmed that LPS stimulation enhanced the binding of ZNF24 to the *MIF* promoter (Fig. 6S–T). Collectively, these findings demonstrate that epithelial cell injury induces ROS accumulation, which promotes ZNF24 expression and subsequently enhances transcriptional activation and secretion of MIF. The released MIF then activates CD74 on macrophages in a paracrine manner, thereby driving M1 polarization and proinflammatory cytokine production.

### 3.7 Pharmacological inhibition of NF-κB mitigates MIF-CD74-driven inflammation and M1 polarization in chronic prostatitis

**Fig. 7:**
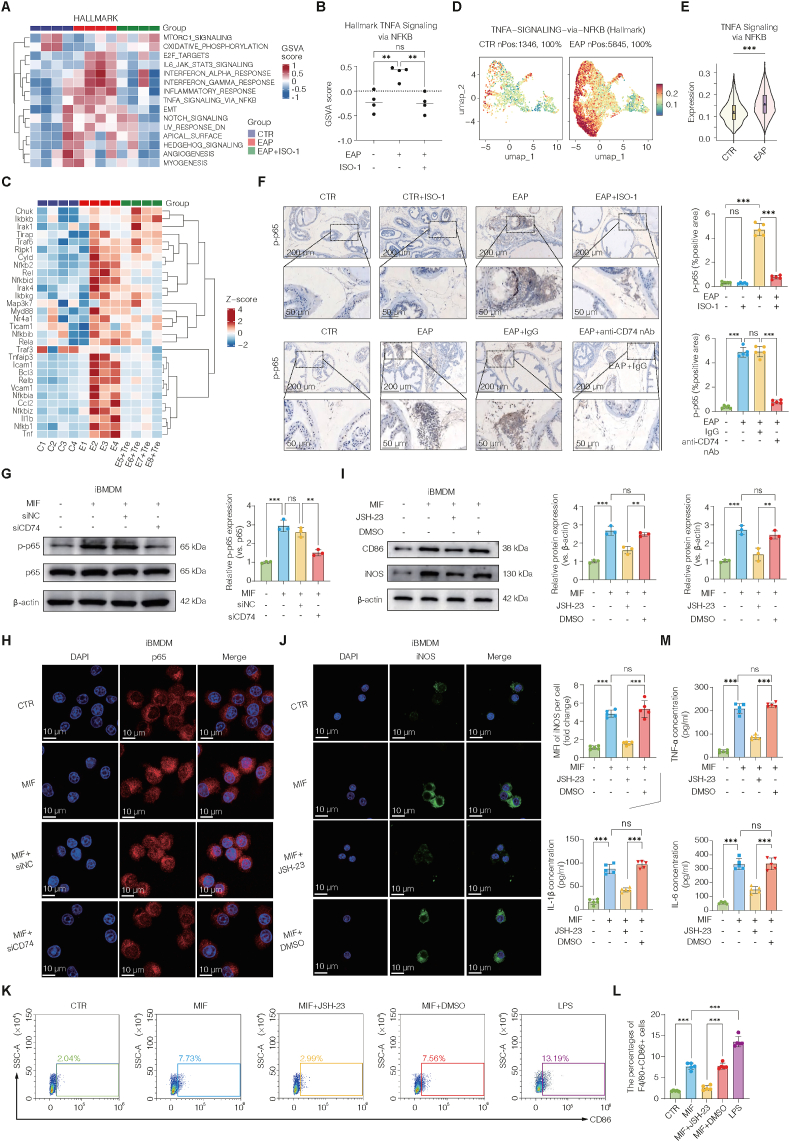
Pharmacological inhibition of NF-κB mitigates MIF-CD74-driven inflammation and M1 polarization in chronic prostatitis. (A) Heatmap of GSEA HALLMARK pathways showing increased inflammatory and NF-κB-related signaling in EAP, reversed by ISO-1. (B) Quantification of TNFA signaling via NF-κB scores from HALLMARK enrichment based on RNA-seq results derived from mouse model. (C) Heatmap of key NF-κB pathway genes, upregulated in EAP and suppressed by ISO-1 based on RNA-seq results derived from mouse model. (D–E) Single-cell transcriptomic analysis showing increased NF-κB pathway activity in EAP prostates using AddModuleScore. (F) IHC of p-p65 confirming NF-κB activation in EAP and inhibition by ISO-1 or CD74 blockade. (G) *In vitro* validation in iBMDMs: MIF-induced p65 phosphorylation reversed by si*CD74*. (H) Confocal immunofluorescence showing MIF-induced nuclear translocation of p65 in iBMDMs, abolished by si*CD74*. (I) Western blot showing suppression of MIF-induced CD86 and iNOS upregulation by the NF-κB inhibitor JSH-23. (J) Confocal staining showing decreased iNOS after JSH-23 treatment in MIF-stimulated iBMDMs. (K–L) Flow cytometry and quantification of F4/80+CD86^+^ macrophages showing reduced M1 polarization with JSH-23. (M) ELISA showing suppression of IL-6, IL-1β, and TNF-α secretion by JSH-23. Data are presented as the mean ± SD. ns, not significant; ∗p < 0.05; ∗∗p < 0.01; ∗∗∗p < 0.001. **Abbreviations:** GSEA, Gene Set Enrichment Analysis; HALLMARK, curated gene set collection; AddModuleScore, single-cell pathway activity scoring method; JSH-23, NF-κB inhibitor.

To identify signaling pathways downstream of MIF that contribute to the pathogenesis of CNP, bulk transcriptomic profiling was conducted on prostate tissues collected from control, CNP, and ISO-1-treated EAP mice. Volcano plots revealed substantial transcriptional reprogramming in both comparisons: CNP vs. control and ISO-1-treated CNP vs. untreated EAP mice (Fig. S9A–B). Gene Set Enrichment Analysis (GSEA, HALLMARK) identified a broad activation of inflammatory signaling cascades, encompassing the interferon-α and interferon-γ responses, inflammatory reaction-related pathways, and notably, the NF-κB signaling pathway, in EAP mice (Fig. 7A). ISO-1 treatment effectively reversed this activation, particularly the NF-κB signature (Fig. 7B–C).

Consistent with these findings, scRNA-seq analysis combined with the AddModuleScore further confirmed enhanced NF-κB pathway activity at the single-cell level in CNP prostates compared with controls (Fig. 7D–E). To validate these transcriptional observations, we performed Western blot analysis of prostate tissues, which revealed that both ISO-1- and anti-CD74-neutralizing antibodies significantly suppressed NF-κB pathway activation in EAP mice (Fig. S9C). These findings were further corroborated by immunohistochemical staining, which revealed decreased p65 nuclear localization and overall NF-κB activity following MIF/CD74 inhibition (Fig. 7F).

To directly investigate the mechanistic role of NF-κB signaling downstream of MIF-CD74 *in vitro*, we employed the iBMDM macrophage line and treated the cells with recombinant MIF. MIF stimulation induced robust activation of the NF-κB pathway, as evidenced by increased phosphorylation and nuclear translocation of the p65 subunit (Fig. 7G–H). This activation was abrogated by CD74 knockdown via siRNA (siCD74), confirming that MIF-mediated NF-κB activation is dependent on CD74. Furthermore, Western blot analysis indicated that MIF treatment significantly upregulated the expression of the M1-associated markers CD86 and iNOS, both of which were markedly suppressed by pretreatment with the NF-κB inhibitor JSH-23 (Fig. 7I). Consistent with these findings, confocal immunofluorescence analysis revealed that MIF-induced iNOS expression was also reversed upon JSH-23 treatment (Fig. 7J).

Flow cytometry further demonstrated that MIF enhanced M1 macrophage polarization, which was significantly reduced upon JSH-23 treatment (Fig. 7K–L). At the molecular level, MIF-induced expression of the proinflammatory cytokines *Il6*, *Il1b*, and *Tnf* was significantly suppressed by JSH-23, as confirmed by both qRT‒PCR and ELISA (Fig. 7M, and Fig. S9D). Collectively, these results demonstrate that MIF promotes prostate inflammation by activating the NF-κB pathway via CD74, thereby enhancing M1 macrophage polarization and proinflammatory cytokine release. Targeting this axis with either CD74 blockade or NF-κB inhibition markedly mitigates inflammation, highlighting a potential therapeutic strategy for chronic prostatitis.

### 3.8 MIF promotes NF-κB activation through the CD74-PKM2 interaction and nuclear translocation

**Fig. 8:**
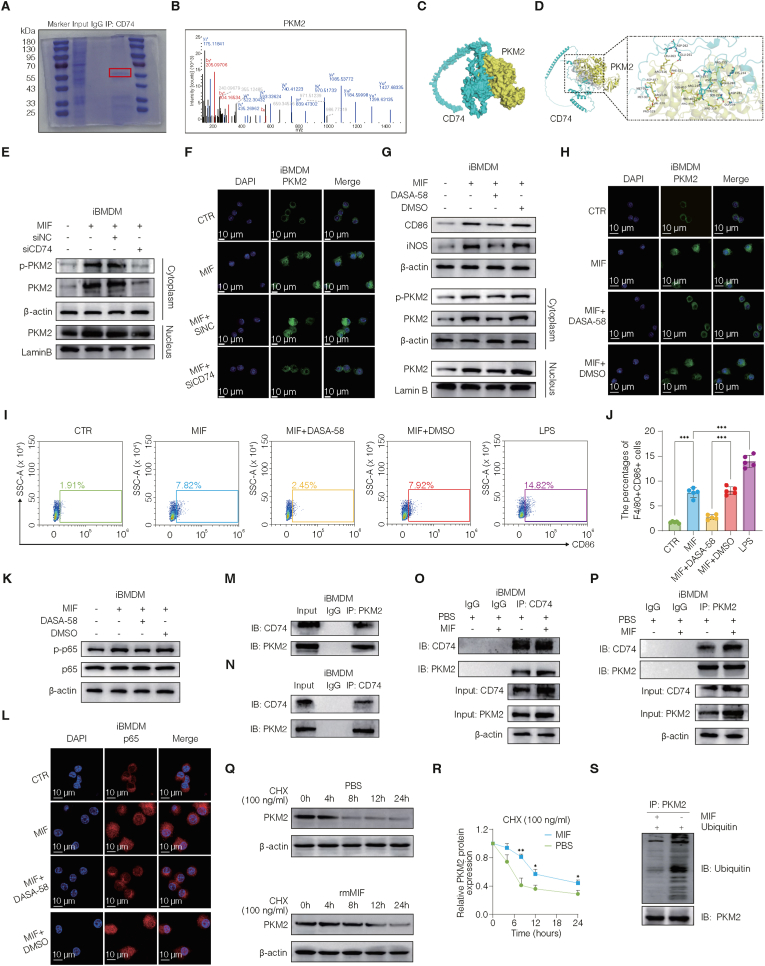
MIF promotes NF-κB activation through CD74-PKM2 interaction and PKM2 nuclear translocation. (A–D) Identification of PKM2 as a CD74-interacting protein in MIF-stimulated iBMDMs, demonstrated by Coomassie-stained SDS-PAGE and mass spectrometry (A–B), and supported by AlphaFold3 structural modeling of the CD74-PKM2 interaction interface (C–D). (E–F) MIF induces PKM2 expression and nuclear accumulation in iBMDMs, which is abolished by CD74 knockdown, as shown by subcellular fractionation and confocal imaging. (G–L) Pharmacological stabilization of PKM2 tetramers with DASA-58 suppresses MIF-induced PKM2 phosphorylation and nuclear localization (G–H), reduces M1 macrophage polarization (I–J), and attenuates NF-κB activation, as indicated by reduced p65 phosphorylation and nuclear translocation (K–L). (M–P) Reciprocal co-immunoprecipitation assays confirming endogenous CD74-PKM2 interaction and its enhancement upon MIF stimulation. (Q–R) Cycloheximide chase assays showing that MIF increases PKM2 protein stability in iBMDMs. (S) *In vitro* ubiquitination assay demonstrating reduced PKM2 ubiquitination following MIF treatment. Data are presented as mean ± SD. ns, not significant; ∗p < 0.05; ∗∗p < 0.01; ∗∗∗p < 0.001. **Abbreviations:** DASA-58, PKM2 tetramer stabilizer; CHX, cycloheximide; SD, standard deviation.

To dissect the mechanism by which MIF-CD74 signaling activates NF-κB, we first performed co-immunoprecipitation of CD74 in iBMDM cells stimulated with recombinant MIF. Coomassie staining revealed a differential band at ∼55–70 kDa compared with that of the IgG control (Fig. 8A), which was identified by mass spectrometry as PKM2 together with several other candidates, including MYH9, RPL8, UBA52, and RPS3A (Fig. 8B, and Fig. S10A). PKM2, as a glycolytic enzyme, regulates cellular metabolism and translocates into the nucleus as a transcriptional co-activator, suggesting its potential cooperation with CD74 in linking metabolic regulation and inflammatory signaling. In support of this hypothesis, AlphaFold3-based structural modeling predicted a high-confidence interaction between PKM2 and CD74 (Fig. 8C–D).

Consistent with this prediction, MIF stimulation increased PKM2 protein levels, whereas siRNA-mediated CD74 knockdown markedly reduced PKM2 abundance in both the cytoplasmic and nuclear compartments (Fig. 8E, and Fig. S10B). Immunofluorescence further confirmed that MIF promoted PKM2 expression and nuclear localization, both of which were abrogated by CD74 silencing (Fig. 8F). Functionally, pharmacological activation of PKM2 with DASA-58, which stabilizes its tetrameric metabolic form and prevents nuclear entry, reversed MIF-induced upregulation of CD86 and iNOS (Fig. 8G, upper panel, and Fig. S10C). DASA-58 also suppressed PKM2 and *p*-PKM2 in both the cytoplasmic and nuclear fractions (Fig. 8G, lower panel, and Fig. S10C) and diminished MIF-driven PKM2 nuclear accumulation and iNOS expression, as shown by confocal microscopy (Fig. 8H, and Fig. S10D). Flow cytometry demonstrated that DASA-58 significantly inhibited MIF-induced M1 polarization (Fig. 8I–J). At the molecular level, DASA-58 blocked MIF-induced IL-1β, IL-6, and TNF-α expression, as measured by ELISA (Fig. S10E), and reduced NF-κB activation, as evidenced by decreased p65 phosphorylation and impaired nuclear translocation (Fig. 8K–L and Fig. S10F).

To directly verify this interaction, reciprocal co-immunoprecipitation assays were used to confirm that CD74-PKM2 binding was markedly enhanced upon MIF stimulation (Fig. 8M–P). Moreover, cycloheximide chase experiments revealed that MIF treatment significantly increased PKM2 stability (Fig. 8Q–R). Co-immunoprecipitation further revealed that MIF reduced PKM2 ubiquitination, suggesting posttranslational stabilization (Fig. 8S). Together, these findings demonstrate that MIF promotes the formation of a CD74-PKM2 complex, which facilitates PKM2 nuclear translocation and activates NF-κB signaling.

### 3.9 MIF-CD74 signaling drives glycolytic reprogramming and mitochondrial dysfunction in M1 macrophages

**Fig. 9:**
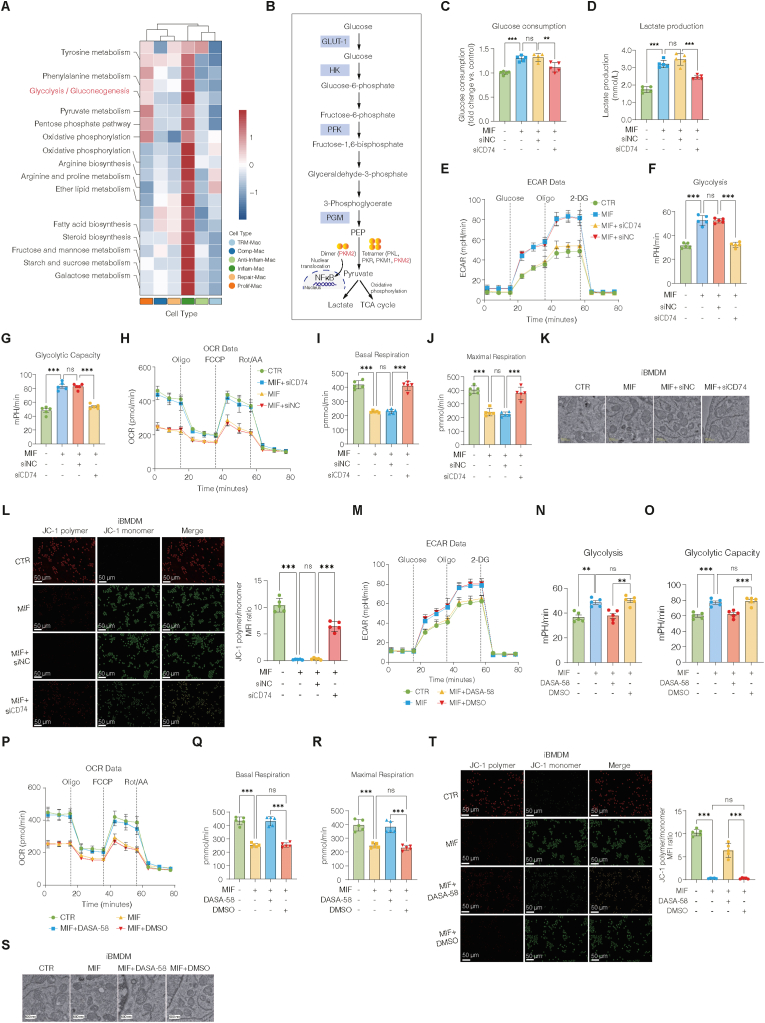
MIF-CD74 signaling drives glycolytic reprogramming and mitochondrial dysfunction in M1 macrophages. (A) Heatmap of the results of the metabolic pathway analysis showing enrichment in inflammatory macrophages, with glycolysis/gluconeogenesis being most prominently upregulated. (B) Schematic of the glycolytic pathway highlighting the role of PKM2 in linking glycolysis to the TCA cycle and inflammatory signaling. (C–D) Glucose consumption and lactate production in iBMDMs with MIF-induced metabolic activation were reversed by si*CD74*. (E–G) ECAR analysis showing increased glycolytic flux and capacity after MIF stimulation, which was suppressed by si*CD74*. (H–J) OCR analysis showing reduced basal and maximal respiration in MIF-treated cells, which was restored by si*CD74*. (K) Transmission electron microscopy image showing fragmented mitochondria with sparse cristae in MIF-treated macrophages, which were rescued by si*CD74*. (L) JC-1 staining showing the loss of the Δψm upon MIF stimulation, which was reversed by si*CD74*. (M–O) ECAR analysis showing the suppression of MIF-driven glycolysis by DASA-58. (P–R) OCR analysis showing recovery of basal and maximal respiration with DASA-58. (S) Transmission electron microscopy confirmed that DASA-58 alleviated MIF-induced mitochondrial fragmentation and cristae loss. (T) JC-1 staining showing the rescue of MIF-induced mitochondrial depolarization by DASA-58. The data are presented as the means ± SD. ns, not significant; ∗p < 0.05; ∗∗p < 0.01; ∗∗∗p < 0.001. **Abbreviations:** scMetabolism, single-cell metabolic pathway analysis; ECAR, extracellular acidification rate; OCR, oxygen consumption rate; Δψm, mitochondrial membrane potential.

Metabolic reprogramming is a defining feature of M1 macrophage polarization. To explore the role of MIF-CD74 signaling, we performed scMetabolism analysis of single-cell RNA-seq data from EAP prostates. Inflammatory macrophages exhibited broad activation of metabolic pathways, with glycolysis/gluconeogenesis being most prominently enriched (Fig. 9A). Pathway mapping highlighted PKM2 as a glycolytic enzyme linking glucose metabolism to TCA cycle entry and inflammatory signaling (Fig. 9B). Consistently, heatmap analysis revealed the upregulation of glycolytic genes in inflammatory macrophages from EAP mice (Fig. S11A).

*In vitro*, MIF stimulation significantly increased glucose consumption and lactate secretion in iBMDMs, and these effects were abolished by CD74 knockdown (Fig. 9C–D). Seahorse extracellular acidification rate (ECAR) assays confirmed enhanced glycolytic flux and capacity upon MIF treatment, which were suppressed by siCD74 (Fig. 9E–G). Conversely, oxygen consumption rate (OCR) analysis revealed that MIF reduced basal and maximal respiration, indicating impaired mitochondrial oxidative capacity, whereas siCD74 restored mitochondrial function (Fig. 9H–J). Ultrastructural analysis via electron microscopy revealed fragmented mitochondria with sparse cristae in MIF-treated macrophages, which were rescued by siCD74 (Fig. 9K). In line with these findings, JC-1 staining revealed that MIF markedly reduced the Δψm, whereas CD74 knockdown restored Δψm integrity (Fig. 9L).

We next assessed the role of PKM2 via DASA-58, which stabilizes the tetrameric metabolic form of PKM2 and prevents its nuclear translocation. DASA-58 reversed the MIF-induced increases in glucose uptake and lactate release (Fig. S11B–C). ECAR assays demonstrated that DASA-58 suppressed MIF-driven glycolytic activation (Fig. 9M–O), whereas OCR analysis revealed recovery of basal and maximal respiration suppressed by MIF (Fig. 9P–R). Furthermore, electron microscopy and JC-1 staining confirmed that DASA-58 alleviated fragmented mitochondria, sparse cristae, and Δψm depolarization caused by MIF (Fig. 9S–T).

**Fig. 10:**
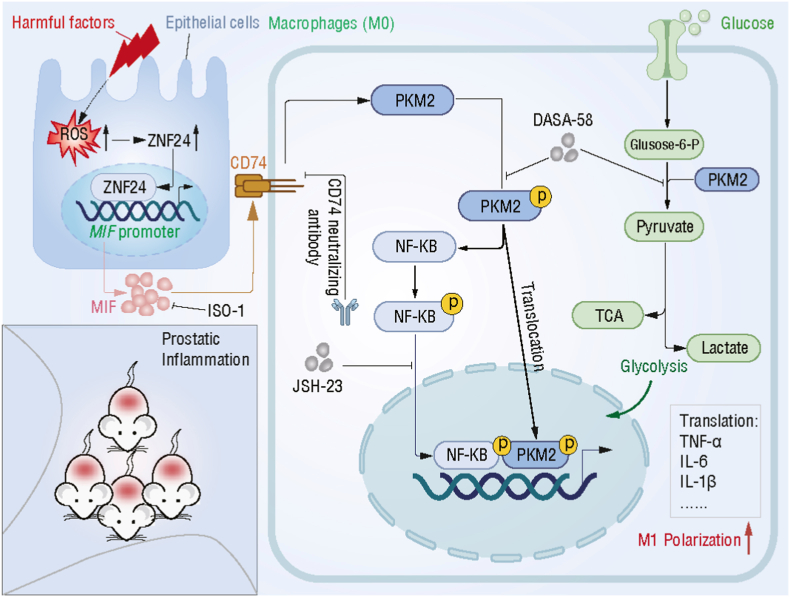
**Schematic illustration of the epithelial ROS-ZNF24-MIF-CD74-PKM2-NF-κB signaling axis in chronic prostatitis.** In response to epithelial injury or inflammatory stimuli, excessive ROS accumulate in prostatic epithelial cells, leading to the activation of the redox-responsive transcription factor ZNF24. Activated ZNF24 directly binds to the MIF promoter and drives MIF transcription, resulting in enhanced MIF production and release. Epithelial-derived MIF subsequently acts in a paracrine manner by binding to CD74 on macrophages. This interaction promotes the stabilization and nuclear translocation of PKM2, which acts as a co-activator to increase NF-κB signaling via p65 nuclear translocation. PKM2 activation also enhances glycolytic flux and contributes to mitochondrial dysfunction, further promoting M1 macrophage polarization and inflammatory cytokine production. Disruption of this axis via ISO-1, CD74 blockade, or PKM2 modulation (DASA-58) effectively mitigates chronic inflammation in the prostate.

Our findings demonstrate that inflammatory stimuli induce excessive ROS accumulation in prostatic epithelial cells, thereby activating the redox-responsive transcription factor ZNF24 to directly drive MIF transcription. The resulting epithelial-derived MIF promotes chronic prostatic inflammation by enhancing glycolytic reprogramming and inducing M1 macrophage polarization via the CD74-PKM2-NF-κB signaling axis. Both pharmacological and genetic inhibition of this pathway significantly attenuated prostatic inflammation in the EAP mouse model (Fig. 10).

## 4 Discussion

CNP remains a major clinical challenge due to its multifactorial pathogenesis, frequent relapse, and limited treatment options [49,50]. In this study, we identify epithelial-derived MIF as a key effector linking epithelial redox stress to macrophage immunometabolism reprogramming in CNP. We show that inflammatory injury induces robust ROS accumulation within prostatic epithelium, which in turn activates the redox-responsive transcription factor ZNF24. ZNF24 directly binds the MIF promoter, driving MIF transcription and secretion in a ROS-dependent manner. Downstream of this epithelial redox axis, epithelial cell-derived MIF drives macrophage M1 polarization via the MIF‒CD74 axis while promoting the CD74‒PKM2 interaction to increase glycolysis and reinforce inflammatory programming. Pharmacological modulation of PKM2 counteracts MIF-driven M1 polarization, and NF-κB activation has been identified as a downstream effector of this cascade. Together, these findings establish a redox-initiated epithelial ROS-ZNF24-MIF-CD74-PKM2-NF-κB cascade as a central pathway sustaining chronic prostatic inflammation.

MIF is a pleiotropic proinflammatory cytokine that exerts broad immunoregulatory effects and plays a pivotal role in regulating immune and inflammatory responses, cellular proliferation, tumorigenesis, and the counterregulatory actions of glucocorticoids [51]. Numerous studies have implicated MIF in the pathogenesis of diverse autoimmune and inflammatory diseases [[52], [53], [54]]. For example, elevated concentrations of MIF have been reported in the serum of patients with ulcerative colitis and Crohn's disease and are positively correlated with disease activity [55,56]; likewise, rheumatoid arthritis patients present increased MIF in blood, synovial fluid and inflamed synovial tissues [57].

Consistent with these findings, our study demonstrates that epithelial-derived MIF is markedly upregulated in both CNP patients and EAP mice and serves as a pivotal paracrine signal that drives macrophage M1 polarization and proinflammatory cytokine production within the prostatic microenvironment. This parallels the findings of Meyer-Siegler et al. [58], who demonstrated constitutive MIF expression and secretion by prostate epithelial cells under chronic environmental injury, thereby sustaining local inflammation. It is worth noting that the release of TNF-α and IL-6 induced by MIF does not always have a pathological effect in all situations. In the early stage of inflammation, the moderately elevated levels of TNF-α and IL-6 help to promote the recruitment of immune cells, enhance the innate immune response, and by activating inflammatory signaling pathways, limit the initial tissue damage and pathogen spread, thereby exerting certain host defense functions [59,60]. However, when the inflammatory stimulus persists, MIF, as an inflammatory amplification factor, its continuous high expression can drive macrophages to shift towards the inflammatory M1 phenotype, leading to the long-term high-level release of TNF-α and IL-6. This uncontrolled pro-inflammatory response may exceed the protective threshold of the host defense, gradually evolving into immune overactivation and ultimately inducing pathological tissue damage in the immune system. In this study, we observed that the expression level of MIF was significantly increased in prostate tissues of CNP patients and in the EAP mouse model, accompanied by an increase in the proportion of M1-type macrophages and the continuous upregulation of TNF-α and IL-6. This suggests that in the context of chronic inflammation, excessive MIF may promote the inflammatory polarization of macrophages, maintain and amplify the abnormal release of pro-inflammatory factors, thereby exacerbating the local inflammatory response in the prostate and promoting the progression of tissue damage.

Although prostate epithelial cells are capable of releasing inflammatory mediators in response to *Trichomonas vaginalis* infection [61,62], their specific role in CNP has remained undefined. We further showed that LPS stimulation markedly upregulated MIF expression and secretion in prostate epithelial cells. At the mechanistic level, we identify ZNF24 as a redox-responsive transcription factor that directly couples epithelial ROS accumulation to MIF transcriptional activation. Under inflammatory stress, excessive ROS accumulation in epithelial cells promotes the upregulation and promoter occupancy of the redox-responsive transcription factor ZNF24, thereby directly driving MIF gene transcription. Consistently, pharmacological scavenging of ROS or genetic silencing of ZNF24 markedly suppresses epithelial MIF expression and secretion, identifying the ROS-ZNF24 axis as a critical upstream regulatory pathway controlling MIF production under inflammatory conditions. These findings position ZNF24 as a previously unrecognized redox-sensitive regulator of MIF and provide a mechanistic link between epithelial oxidative stress and macrophage-directed inflammatory signaling. Meanwhile, our research results indicate that ROS in epithelial cells cannot directly regulate macrophage polarization, as in the co-culture system of LPS-stimulated primary prostate epithelial cells and iBMDM, even when ROS still exists, specific inhibition of MIF signaling can significantly weaken the M1 polarization of macrophages. This suggests that ROS alone is not sufficient to independently maintain the pro-inflammatory phenotype of immune cells, but needs to exert its immune regulatory function through the secretory factor of MIF. It may be because ROS has high reactivity and temporariness, and its main function lies in regulating the stress response and transcriptional program within epithelial cells, thereby inducing the production of inflammatory mediators. In contrast, MIF, as a stable secreted cytokine, can persist in the tissue microenvironment and directly act on immune cells through CD74-mediated signal transduction, driving macrophages to polarize towards the inflammatory M1 phenotype and maintaining the amplification loop of pro-inflammatory factors. Despite numerous studies have highlighted the essential contribution of MIF to diverse inflammatory diseases [[63], [64], [65]], its specific role in CNP has not been investigated. Accordingly, we assessed the impact of MIF inhibition on CNP progression and found that pharmacological blockade of MIF significantly alleviated prostatic inflammation and pain sensitivity in EAP model mice. Given that CD74 serves as the cognate receptor of MIF, we hypothesized that MIF signals via CD74; indeed, the administration of anti-CD74 neutralizing antibodies to EAP mice reduced both prostatic inflammation and nociceptive responses, confirming the involvement of the MIF‒CD74 axis in CNP pathogenesis.

Macrophages are widely recognized as central players in the pathogenesis and progression of CNP, with M1-polarized macrophages driving inflammatory responses and exacerbating disease severity [13,66]. Previous studies have indicated that MIF plays a critical role in macrophage development, differentiation, and polarization [67]. Our data demonstrate that MIF promotes M1 polarization in macrophages, and *in vitro* co-culture assays confirmed that prostate epithelial cell-derived MIF enhances M1 polarization; conversely, CD74 blockade attenuates MIF-induced M1 skewing. These findings implicate the MIF/CD74 axis in chronic prostatitis onset and progression via the induction of M1 macrophage polarization. RNA sequencing of prostatic tissues from EAP mice revealed robust activation of the NF-κB pathway, which tightly correlates with M1 macrophage polarization. As a pivotal signaling cascade in immune and inflammatory responses, NF-κB activation typically accompanies proinflammatory cytokine release and immune-cell polarization [33]. However, the mechanistic link between MIF-driven M1 polarization and NF-κB activation remains largely unexplored. To address this, we stimulated macrophages with MIF, performed CD74 pull-down followed by mass spectrometry, and identified PKM2, which was previously implicated in immune inflammatory responses [29]. As a member of the pyruvate kinase family, PKM2 mediates the conversion of phosphoenolpyruvate and ADP to pyruvate and ATP, thereby regulating glycolytic flux.

Extensive evidence indicates that cells bolster glucose utilization by upregulating glycolysis, a metabolic program that supports inflammatory responses by driving immune-cell activation and promoting oxidative inactivation [68,69]. In macrophages, PKM2 expression governs not only glycolytic flux but also, through its regulatory interactions with multiple signaling cascades, macrophage polarization and immune function [70]. For example, Rao et al. [33] demonstrated that PKM2 enhances M1 polarization and the release of proinflammatory cytokines (IL-6, TNF-α, and IL-1β), thereby amplifying inflammation, whereas shikonin attenuates ulcerative colitis by inhibiting PKM2 nuclear translocation and suppressing M1 commitment [71]. PKM2 exists in low-activity monomeric/dimeric forms, which translocate to the nucleus to act as transcriptional coactivators for glycolytic and inflammatory genes (e.g., *GLUT1*, *LDHA*, *HK2*), and in high-activity tetrameric forms, predominantly in the cytoplasm, which favors tricarboxylic acid cycling, oxidative phosphorylation and anti-inflammatory responses [72,73]. Phosphorylation at Tyr^105^ modulates the monomer‒tetramer equilibrium, thereby balancing glycolysis with oxidative metabolism [74,75]. The transition from tetramers to monomers/dimers enhances the glycolytic capacity and M1 polarization of macrophages. In our study, MIF stimulation augmented macrophage glycolysis and promoted both the cytosolic accumulation and nuclear translocation of PKM2. To dissect this mechanism, we employed DASA-58, a small-molecule PKM2 activator that stabilizes the tetrameric conformation and diminishes glycolytic and pentose phosphate pathway intermediates [76]. DASA-58 markedly reversed MIF-induced glycolytic skewing, M1 polarization and NF-κB activation in macrophages. Moreover, the inhibition of NF-κB with JSH-23 significantly attenuated MIF-driven M1 polarization. Collectively, these findings reveal that the MIF‒CD74 axis drives M1 macrophage polarization in CNPs by promoting PKM2-dependent glycolysis and downstream NF-κB signaling. To our knowledge, this is the first report implicating an MIF-CD74-PKM2-glycolytic cascade in the pathogenesis of prostatitis, providing new molecular insights into CNP and highlighting MIF as a promising therapeutic target.

## 5 Conclusion

In summary, we identified a critical epithelial-macrophage crosstalk mechanism in CNP in which epithelial oxidative stress serves as the initiating event that activates a ZNF24-MIF transcriptional program, which in turn drives CD74-dependent PKM2-NF-κB signaling and glycolytic reprogramming to reinforce M1 macrophage polarization and sustain proinflammatory cytokine production. Pharmacological or genetic blockade of this axis effectively alleviated chronic prostatic inflammation and pain in EAP mice, underscoring its translational potential. This epithelial ROS-ZNF24-MIF-CD74 signaling axis provides a mechanistic framework linking redox stress to chronic immunometabolic inflammation and highlights multiple therapeutic entry points for precision intervention in CNP.

## Ethics statement

The Ethics Committee of the First Affiliated Hospital of Anhui Medical University approved the patient recruitment protocol (PJ-2020-07-11, PJ-2021-03-13). All animal experiments were authorized by the Animal Care and Use Committee of Anhui Medical University's Animal Center (LLSC20241750).

## Availability of data and materials

The datasets generated and/or analyzed during the current study are available from the corresponding author upon reasonable request. .

## Consent for publication

All the authors have read and approved the final manuscript and consent to its publication.

## Funding

This work is supported by the 10.13039/501100001809National Natural Science Foundation of China (82470800 & 82570900), the 10.13039/501100003995Natural Science Foundation of Anhui Province (2308085MH247), the Research Fund of Anhui Institute of Translational Medicine (2022zhyx-B13), and the Anhui Province Higher Education Science Research Project Outstanding Youth Research Project (2024AH030029).

## CRediT authorship contribution statement

**Fei Zhang:** Conceptualization, Data curation, Formal analysis, Investigation, Methodology, Software, Writing – original draft. **Andong Zhang:** Data curation, Methodology, Software, Writing – original draft. **Tong Meng:** Data curation, Investigation, Writing – original draft. **Xianhong Liu:** Formal analysis, Methodology, Validation. **Cheng Yang:** Conceptualization, Methodology, Writing – review & editing. **Chaozhao Liang:** Conceptualization, Data curation, Writing – review & editing. **Meng Zhang:** Conceptualization, Funding acquisition, Investigation, Methodology, Resources, Supervision, Validation, Visualization, Writing – review & editing.

## Declaration of competing interest

The authors declare that they have no known competing financial interests or personal relationships that could have appeared to influence the work reported in this paper.

## Data Availability

Data will be made available on request.

## Appendix A Supplementary data

Fig. S1 **Elevated epithelial MIF expression associates with prostate inflammation severity.** (A) Quantification of MIF-positive areas in prostate tissues from BPH patients with mild, moderate, or severe inflammation. (B) Stacked area plot showing the relative proportions of major cell types across individual samples. (C) Quantification of MIF fluorescence intensity within the EPCAM^+^ epithelial compartment of BPH prostate tissues with increasing inflammation severity. (D) Quantification of MIF-positive areas in prostate tissues from control (CTR) and EAP mice. (E) Representative immunofluorescence (IF) images of mouse prostate sections showing co-localization of MIF (green) and EPCAM (red) in the prostatic epithelium of EAP mice. (F) Quantification of MIF fluorescence intensity within the EPCAM^+^ epithelial regions in prostate tissues from CTR and EAP mice. Data are presented as mean ± SD. ns, not significant; ∗p < 0.05; ∗∗p < 0.01; ∗∗∗p < 0.001. **Abbreviations:** CTR, control; EAP, experimental autoimmune prostatitis. Fig. S1

Fig. S2 Quality control metrics for the single-cell RNA-seq data. Violin plots displaying UMI counts, detected genes, and proportions of mitochondrial, ribosomal, and hemoglobin transcripts across six mouse prostate samples. Fig. S2

Fig. S3 Cell type annotation and functional characterization of mouse prostate single-cell RNA-seq data. (A) UMAP plots showing representative marker genes used to define major cell types, including epithelial (*Epcam*, *Krt18*), stromal (*Col1a1*), and immune populations (*Cd3e*, *Cd79a*, *Itgax*, *Cd68*), together with other lineage-specific markers. (B) Heatmap of marker gene expression (Z-score normalized) across major and secondary cell types, illustrating cell-type specificity and hierarchical relationships. (C) Functional enrichment analysis of cell type-specific genes, displaying representative genes, associated Gene Ontology biological process (GO_BP) terms, and functional annotations (e.g., immune activation, epithelial differentiation, and extracellular matrix remodeling). (D) Dot plots showing incoming (top) and outgoing (bottom) intercellular communication probabilities across major cell types, revealing prominent epithelial-to-macrophage signaling via MIF. (E) Network diagrams depicting the number (top) and strength (bottom) of inferred intercellular interactions among cell types. (F) Spatial distribution map showing Cell2location-inferred macrophage-associated spatial signals across prostate tissue sections. Regions enriched for MIF- and CD74-related expression patterns are indicated by increased signal intensity. Each spot represents estimates derived from spatial transcriptomics data. Fig. S3

Fig. S4 Cell2location model training, reconstruction accuracy, and spatial mapping. (A) Training dynamics of cell2location showing the decline in negative evidence lower bound (-ELBO) loss across 10,000 epochs, indicating rapid optimization and convergence. (B) Reconstruction accuracy of the trained model, comparing posterior expected values with observed data (log10-transformed). The alignment along the diagonal reflects robust recovery of gene expression distributions. (C) Spatial distribution of representative epithelial (basal, intermediate, and luminal) and immune (macrophage) cell types inferred by cell2location, illustrating distinct tissue localization patterns. Fig. S4

Fig. S5 **Macrophage subpopulation distribution and M1-associated gene expression in CTR and EAP.** (A) Frequencies of macrophage subpopulations in CTR and EAP. (B) UMAP feature plots of M1-associated genes (*Nos2*, *Il1b*, *Tnf*, *Cd86*) in CTR and EAP. Data are presented as the mean ± SD. ns, not significant. **Abbreviations:** CTR, control; EAP, experimental autoimmune prostatitis. Fig. S5

Fig. S6 **ISO-1 treatment alleviates EAP-induced prostatic inflammation and downregulates CD74 expression *in vivo*.** (A) Experimental timeline: CNP was induced by EAP (dual immunization), and ISO-1 (3.5 mg/kg, intraperitoneal injection) was administered daily for 2 weeks before tissue collection on day 42. (B) qRT‒PCR analysis showing suppression of *Tnf*, *Il1b*, and *Il6* expression in prostate tissues. (C) IHC of CD74 in prostate tissues showing decreased expression with ISO-1. (D) Western blot analysis of CD74 protein in ISO-1-treated versus untreated EAP mice. Data are presented as the mean ± SD. ns, not significant; ∗p < 0.05; ∗∗p < 0.01; ∗∗∗p < 0.001. **Abbreviations:** CTR, control; EAP, experimental autoimmune prostatitis. Fig. S6

Fig. S7 Blockade of CD74 suppresses pro-inflammatory cytokine expression in EAP-induced prostate tissues. (A) qRT‒PCR analysis of *Tnf*, *Il6*, and *Il1b* expression in mouse prostate tissues, which was suppressed by the CD74 nAb. The data are presented as the means ± SD. ns, not significant; ∗p < 0.05; ∗∗p < 0.01; ∗∗∗p < 0.001. **Abbreviations:** CD74 nAb, anti-CD74 neutralizing antibodies; CTR, control; EAP, experimental autoimmune prostatitis; qRT‒PCR, quantitative real-time PCR. Fig. S7

Fig. S8 An epithelial ROS–ZNF24–MIF axis promotes CD74-dependent macrophage M1 polarization. (A–B) Dose- and time-dependent induction of CD86 and iNOS in iBMDMs following MIF stimulation. (C–F) CD74 knockdown in iBMDMs suppresses MIF-induced M1 marker expression and proinflammatory cytokine production, as shown by western blot (C–D), iNOS immunofluorescence quantification (E), and qRT–PCR analysis of *Il1b*, *Il6*, and *Tnf* (F). (G) Immunofluorescence showing increased epithelial MIF expression in RWPE-1 cells after LPS stimulation. (H) ROS scavenging with NAC suppresses LPS-induced MIF expression and secretion in RWPE-1 cells, as assessed by qRT-PCR (left) and ELISA (right). (I) ZNF24 ChIP-seq peak enrichment at the MIF promoter based on ReMap 2022 datasets. (J–L) LPS-induced ZNF24 upregulation and ZNF24-dependent MIF expression in RWPE-1 cells, as shown by ZNF24 protein quantification (J), *ZNF24* siRNA knockdown efficiency (K), and reduced *MIF* mRNA expression and secretion following ZNF24 silencing (L). Data are presented as mean ± SD. ns, not significant; ∗p < 0.05; ∗∗p < 0.01; ∗∗∗p < 0.001.Abbreviations: NAC, N-Acetylcysteine; ROS, Reactive Oxygen Species. Fig. S8

Fig. S9 **Pharmacological inhibition of MIF or CD74 attenuates NF-κB activation and inflammatory signaling in EAP.** (A-B) Volcano plots of differentially expressed genes in prostate tissues of EAP versus CTR (A) and ISO-1-treated versus untreated EAP (B). (C) Western blot of phosphorylated p65 (p-p65) in prostate tissues, elevated in EAP and reduced by ISO-1 or CD74 blockade. (D) qRT‒PCR showing that JSH-23 reverses MIF-induced *Il6*, *Il1b*, and *Tnf* transcription in iBMDMs. Data are presented as the mean ± SD. ns, not significant; ∗p < 0.05; ∗∗p < 0.01; ∗∗∗p < 0.001.Abbreviations: MIF, macrophage migration inhibitory factor; CTR, control; EAP, experimental autoimmune prostatitis; JSH-23, NF-κB inhibitor; DMSO, dimethyl sulfoxide. Fig. S9

Fig. S10 **MIF triggers NF-κB signaling through CD74-mediated PKM2 nuclear import.** (A) Table of high-confidence CD74-binding candidates from mass spectrometry, including PKM2. (B) Quantification of PKM2 and p- PKM2 protein levels in iBMDMs. (C) Quantification of CD86, iNOS, PKM2 and p- PKM2 protein levels in iBMDMs. (D) Immunofluorescence showing that DASA-58 blocks MIF-induced iNOS expression. (E) ELISA showing the suppression of MIF-induced IL-1β, IL-6, and TNF-α secretion by DASA-58. (F) Quantification of p-p65 protein levels in iBMDMs. The data are presented as the means ± SD. ns, not significant; ∗p < 0.05; ∗∗p < 0.01; ∗∗∗p < 0.001.Abbreviations: DASA-58, PKM2 tetramer stabilizer; CHX, cycloheximide; SD, standard deviation; DMSO, dimethyl sulfoxide. Fig. S10

Fig. S11 **MIF-CD74 signaling induces metabolic reprogramming toward glycolysis and impairs mitochondrial function in M1 macrophages.** (A) Heatmap of glycolytic gene expression across cell types showing increased *Pkm* in inflammatory macrophages. (B–C) Glucose consumption and lactate production data showing that treatment with DASA-58 reversed MIF-induced glycolytic activation. The data are presented as the means ± SD. ns, not significant; ∗p < 0.05; ∗∗p < 0.01; ∗∗∗p < 0.001.Abbreviations: SD, standard deviation. Fig. S11

## References

[1] Schaeffer A.J. (2006). Chronic prostatitis and the chronic pelvic pain syndrome. N. Engl. J. Med..

[2] Magistro G., Wagenlehner F.M.E., Grabe M., Weidner W., Stief C.G., Nickel J.C. (2016). Contemporary management of chronic Prostatitis/Chronic pelvic pain syndrome. Eur. Urol..

[3] Chung P.H., Swaminathan V., Spigner S.T., Leong J.Y., Bulafka J., Frasso R. (2022). Genitourinary and sexual symptoms and treatments in transfeminine individuals: a qualitative exploration of patients' needs. Sex. Med..

[4] Murray C.B., Li R., Kashikar-Zuck S., Zhou C., Palermo T.M. (2025). Adolescent predictors of young adult pain and health outcomes: results from a 6-year prospective follow-up study. Pain.

[5] Kaplan C.M., Kelleher E., Irani A., Schrepf A., Clauw D.J., Harte S.E. (2024). Deciphering nociplastic pain: clinical features, risk factors and potential mechanisms. Nat. Rev. Neurol..

[6] Ackerman A.L., Torosis M., Jackson N.J., Caron A.T., Kaufman M.R., Lowder J.L. (2023). The Persistency Index: a novel screening tool for identifying myofascial pelvic floor dysfunction in patients seeking care for lower urinary tract symptoms. Am. J. Obstet. Gynecol..

[7] Vasdev N., Thorpe A., Nikibakhsh A.A. (2011). Clinical Management of Complicated Urinary Tract Infection.

[8] Kfoury Y., Baryawno N., Severe N., Mei S., Gustafsson K., Hirz T. (2021). Human prostate cancer bone metastases have an actionable immunosuppressive microenvironment. Cancer Cell.

[9] Jin B.R., Kim H.J., Na J.H., Lee W.K., An H.J. (2024). Targeting benign prostate hyperplasia treatments: AR/TGF-β/NOX4 inhibition by apocynin suppresses inflammation and proliferation. J. Adv. Res..

[10] Jin C., Zhang F., Luo H., Li B., Jiang X., Pirozzi C.J. (2024). The CCL5/CCR5/SHP2 axis sustains Stat1 phosphorylation and activates NF-κB signaling promoting M1 macrophage polarization and exacerbating chronic prostatic inflammation. Cell Commun. Signal..

[11] Zhan M., Xu H., Yu G., Chen Q., Yang R., Chen Y. (2024). Androgen receptor deficiency-induced TUG1 in suppressing ferroptosis to promote benign prostatic hyperplasia through the miR-188-3p/GPX4 signal pathway. Redox Biol..

[12] Kumar Jha P., Aikawa M., Aikawa E. (2024). Macrophage heterogeneity and efferocytosis: beyond the M1/M2 dichotomy. Circ. Res..

[13] Zhang Y., Zhang C., Feng R., Meng T., Peng W., Song J. (2024). CXCR4 regulates macrophage M1 polarization by altering glycolysis to promote prostate fibrosis. Cell Commun. Signal..

[14] Sharma D., Ravi R.N., Abdullah A.D.I., Subramaniyan V. (2025). Therapeutic prospects of modulating TLR4/MAPK/ROS signalling in obesity-associated neuroinflammation. Biomed. Pharmacother..

[15] Liu Y., Sun Y., Kang J., He Z., Liu Q., Wu J. (2022). Role of ROS-Induced NLRP3 inflammasome activation in the Formation of calcium oxalate nephrolithiasis. Front. Immunol..

[16] Liu R., Wang C., Huang D., Che H., Piao Y., Hong C. (2025). Lurongdabu Decoction alleviates mitochondrial damage and preserves nasal mucosal barrier integrity via the ESR1/PI3K/AKT and EGFR/FAK/SRC signaling pathways. J. Ethnopharmacol..

[17] Lu Q., Jiao Y., Wu Z., Wen X., Li C. (2025). Zanthoxylum Nitidum ameliorates intestinal barrier dysfunction and inflammation in TNBS-Induced colitis rats and LPS-Stimulated Caco-2 cells. Faseb j..

[18] Zhang Y., Zhang X., Ren J., Sun H., Yang Z., Wang J. (2025). Methylglyoxal induces oxidative stress and ferroptosis of renal tubular epithelial cells in acute and chronic kidney injury mice. Front. Cell Dev. Biol..

[19] Wang H., Slotabec L., Didik S., Li Z., Leng L., Zhao B. (2024). A small molecule macrophage migration inhibitory factor agonist ameliorates age-related myocardial intolerance to ischemia-reperfusion insults via metabolic regulation. Metab. Clin. Exp..

[20] Calandra T., Roger T. (2003). Macrophage migration inhibitory factor: a regulator of innate immunity. Nat. Rev. Immunol..

[21] Leng L., Chen L., Fan J., Greven D., Arjona A., Du X. (2011). A small-molecule macrophage migration inhibitory factor antagonist protects against glomerulonephritis in lupus-prone NZB/NZW F1 and MRL/lpr mice. J. Immunol..

[22] Chen L., Liu Y., Yue S., Wang H., Chen J., Ma W. (2024). P2X7R modulates NEK7-NLRP3 interaction to exacerbate experimental Autoimmune Prostatitis via GSDMD-mediated prostate epithelial cell pyroptosis. Int. J. Biol. Sci..

[23] Song H., Shen Q., Hu S., Jin J. (2020). The role of macrophage migration inhibitory factor in promoting benign prostatic hyperplasia epithelial cell growth by modulating COX-2 and P53 signaling. Biol Open.

[24] Sajko S., Skeens E., Schinagl A., Ferhat M., Mirkina I., Mayer J. (2024). Redox-dependent plasticity of oxMIF facilitates its interaction with CD74 and therapeutic antibodies. Redox Biol..

[25] Kong X.F., Martinez-Barricarte R., Kennedy J., Mele F., Lazarov T., Deenick E.K. (2018). Disruption of an antimycobacterial circuit between dendritic and helper T cells in human SPPL2a deficiency. Nat. Immunol..

[26] Zerpa-Hernández D.A., García-Chagollán M., Sánchez-Zuno G.A., García-Arellano S., Hernández-Bello J., Hernández-Palma L.A. (2024). Expression of transcriptional factors of T helper differentiation (T-bet, GATA-3, RORγt, and FOXP3), MIF receptors (CD44, CD74, CXCR2, 4, 7), and Th1, Th2, and Th17 cytokines in PBMC from control subjects and rheumatoid arthritis patients. Curr. Mol. Med..

[27] Guan D., Li Y., Cui Y., Zhao H., Dong N., Wang K. (2023). 5-HMF attenuates inflammation and demyelination in experimental autoimmune encephalomyelitis mice by inhibiting the MIF-CD74 interaction. Acta Biochim. Biophys. Sin..

[28] Wallace D.J., Figueras F., Wegener W.A., Goldenberg D.M. (2021). Experience with milatuzumab, an anti-CD74 antibody against immunomodulatory macrophage migration inhibitory factor (MIF) receptor, for systemic lupus erythematosus (SLE). Ann. Rheum. Dis..

[29] Angiari S., Runtsch M.C., Sutton C.E., Palsson-McDermott E.M., Kelly B., Rana N. (2020). Pharmacological activation of pyruvate kinase M2 inhibits CD4(+) T cell pathogenicity and suppresses autoimmunity. Cell Metab..

[30] Hu J., Deng F., Zhao B., Lin Z., Sun Q., Yang X. (2022). Lactobacillus murinus alleviate intestinal ischemia/reperfusion injury through promoting the release of interleukin-10 from M2 macrophages via toll-like receptor 2 signaling. Microbiome.

[31] Zhang F., Meng T., Feng R., Jin C., Zhang S., Meng J. (2024). MIF aggravates experimental autoimmune prostatitis through activation of the NLRP3 inflammasome via the PI3K/AKT pathway. Int. Immunopharmacol..

[32] Cheng W.L., Kao Y.H., Chen Y.C., Lin Y.K., Chen S.A., Chen Y.J. (2020). Macrophage migration inhibitory factor increases atrial arrhythmogenesis through CD74 signaling. Transl. Res. : J. Lab. Clin. Med..

[33] Rao J., Wang H., Ni M., Wang Z., Wang Z., Wei S. (2022). FSTL1 promotes liver fibrosis by reprogramming macrophage function through modulating the intracellular function of PKM2. Gut.

[34] Chen X., Liu G., Yuan Y., Wu G., Wang S., Yuan L. (2019). NEK7 interacts with NLRP3 to modulate the pyroptosis in inflammatory bowel disease via NF-κB signaling. Cell Death Dis..

[35] Chen S., Zhou Y., Chen Y., Gu J. (2018). Fastp: an ultra-fast all-in-one FASTQ preprocessor. Bioinformatics.

[36] Kim D., Langmead B., Salzberg S.L. (2015). HISAT: a fast spliced aligner with low memory requirements. Nat. Methods.

[37] Roberts A., Trapnell C., Donaghey J., Rinn J.L., Pachter L. (2011). Improving RNA-Seq expression estimates by correcting for fragment bias. Genome Biol..

[38] Anders S., Pyl P.T., Huber W. (2015). HTSeq--a Python framework to work with high-throughput sequencing data. Bioinformatics.

[39] McGinnis C.S., Murrow L.M., Gartner Z.J. (2019). DoubletFinder: doublet detection in single-cell RNA sequencing data using artificial nearest neighbors. Cell Syst..

[40] Hu J., Liu F., Zhang J., Yin L., Cao W., Xu W. (2025). Spatially resolved transcriptomic analysis of the adult human prostate. Nat. Genet..

[41] Zheng L., Qin S., Si W., Wang A., Xing B., Gao R. (2021). Pan-cancer single-cell landscape of tumor-infiltrating T cells. Science.

[42] Ma R.Y., Black A., Qian B.Z. (2022). Macrophage diversity in cancer revisited in the era of single-cell omics. Trends Immunol..

[43] Wu Y., Yang S., Ma J., Chen Z., Song G., Rao D. (2022). Spatiotemporal immune landscape of colorectal cancer liver metastasis at single-cell level. Cancer Discov..

[44] Jin S., Plikus M.V., Nie Q. (2025). CellChat for systematic analysis of cell-cell communication from single-cell transcriptomics. Nat. Protoc..

[45] Nutton V. (1971). Velia and the school of Salerno. Med. Hist..

[46] Kleshchevnikov V., Shmatko A., Dann E., Aivazidis A., King H.W., Li T. (2022). Cell2location maps fine-grained cell types in spatial transcriptomics. Nat. Biotechnol..

[47] Porpaczy P., Schmidbauer C.P., Georgopoulos A., Rameis H. (1984). Concentrations of doxorubicin in renal interstitial fluid after peripheral intravenous and intrarenal artery infusion in dogs. J. Urol..

[48] Cao J., Spielmann M., Qiu X., Huang X., Ibrahim D.M., Hill A.J. (2019). The single-cell transcriptional landscape of mammalian organogenesis. Nature.

[49] Murphy A.B., Macejko A., Taylor A., Nadler R.B. (2009). Chronic prostatitis: management strategies. Drugs.

[50] Polackwich A.S., Shoskes D.A. (2016). Chronic prostatitis/chronic pelvic pain syndrome: a review of evaluation and therapy. Prostate Cancer Prostatic Dis..

[51] Sumaiya K., Langford D., Natarajaseenivasan K., Shanmughapriya S. (2022). Macrophage migration inhibitory factor (MIF): a multifaceted cytokine regulated by genetic and physiological strategies. Pharmacol. Ther..

[52] Cvetkovic I., Stosic-Grujicic S. (2006). Neutralization of macrophage migration inhibitory factor-novel approach for the treatment of immunoinflammatory disorders. Int. Immunopharmacol..

[53] Kang I., Bucala R. (2019). The immunobiology of MIF: function, genetics and prospects for precision medicine. Nat. Rev. Rheumatol..

[54] Harris J., VanPatten S., Deen N.S., Al-Abed Y., Morand E.F. (2019). Rediscovering MIF: new tricks for an old cytokine. Trends Immunol..

[55] Ohkawara T., Nishihira J., Takeda H., Hige S., Kato M., Sugiyama T. (2002). Amelioration of dextran sulfate sodium-induced colitis by anti-macrophage migration inhibitory factor antibody in mice. Gastroenterology.

[56] Dambacher J., Staudinger T., Seiderer J., Sisic Z., Schnitzler F., Pfennig S. (2007). Macrophage migration inhibitory factor (MIF) -173G/C promoter polymorphism influences upper gastrointestinal tract involvement and disease activity in patients with Crohn's disease. Inflamm. Bowel Dis..

[57] Mikulowska A., Metz C.N., Bucala R., Holmdahl R. (1997). Macrophage migration inhibitory factor is involved in the pathogenesis of collagen type II-induced arthritis in mice. J. Immunol..

[58] Meyer-Siegler K.L., Iczkowski K.A., Leng L., Bucala R., Vera P.L. (2006). Inhibition of macrophage migration inhibitory factor or its receptor (CD74) attenuates growth and invasion of DU-145 prostate cancer cells. J. Immunol..

[59] Kalliolias G.D., Ivashkiv L.B. (2016). TNF biology, pathogenic mechanisms and emerging therapeutic strategies. Nat. Rev. Rheumatol..

[60] Tanaka T., Narazaki M., Kishimoto T. (2014). IL-6 in inflammation, immunity, and disease. Cold Spring Harbor Perspect. Biol..

[61] Han I.H., Kim J.H., Ryu J.S. (2023). Inflammatory response to Trichomonas vaginalis in the pathogenesis of prostatitis and benign prostatic hyperplasia. Parasites Hosts Dis.

[62] Han I.H., Kim J.H., Jang K.S., Ryu J.S. (2019). Inflammatory mediators of prostate epithelial cells stimulated with Trichomonas vaginalis promote proliferative and invasive properties of prostate cancer cells. Prostate.

[63] Oliver J., Márquez A., Gómez-Garcia M., Martinez A., Mendoza J.L., Vilchez J.R. (2007). Association of the macrophage migration inhibitory factor gene polymorphisms with inflammatory bowel disease. Gut.

[64] Zhao L., Du G.L., Ruze A., Qi H.Z., Zhang C.S., Li Q.L. (2025). Novel function of macrophage migration inhibitory factor in regulating post-infarct inflammation and the therapeutic significance. J. Adv. Res..

[65] Nakamura A., Jo S., Nakamura S., Aparnathi M.K., Boroojeni S.F., Korshko M. (2024). HIF-1α and MIF enhance neutrophil-driven type 3 immunity and chondrogenesis in a murine spondyloarthritis model. Cell. Mol. Immunol..

[66] Meng T., Zhang Y., Wang H., Wu W., Peng W., Yue J. (2025). Irf7 aggravates prostatitis by promoting Hif-1α-mediated glycolysis to facilitate M1 polarization. Cell. Mol. Life Sci. : CM.

[67] Yin C., Cai J., Gou Y., Li D., Tang H., Wang L. (2022). Dynamic changes in human THP-1-derived M1-to-M2 macrophage polarization during Thelazia callipaeda MIF induction. Front. Immunol..

[68] Brandes R.P., Rezende F. (2021). Glycolysis and inflammation: partners in crime. Circ. Res..

[69] Gauthier T., Yao C., Dowdy T., Jin W., Lim Y.J., Patiño L.C. (2023). TGF-β uncouples glycolysis and inflammation in macrophages and controls survival during sepsis. Sci. Signal..

[70] Palsson-McDermott E.M., Curtis A.M., Goel G., Lauterbach M.A.R., Sheedy F.J., Gleeson L.E. (2015). Pyruvate kinase M2 regulates Hif-1α activity and IL-1β induction and is a critical determinant of the warburg effect in LPS-Activated macrophages. Cell Metab..

[71] Huang B., Wang Q., Jiang L., Lu S., Li C., Xu C. (2022). Shikonin ameliorated mice colitis by inhibiting dimerization and tetramerization of PKM2 in macrophages. Front. Pharmacol..

[72] Luo W., Hu H., Chang R., Zhong J., Knabel M., O'Meally R. (2011). Pyruvate kinase M2 is a PHD3-stimulated coactivator for hypoxia-inducible factor 1. Cell.

[73] Xie M., Yu Y., Kang R., Zhu S., Yang L., Zeng L. (2016). PKM2-dependent glycolysis promotes NLRP3 and AIM2 inflammasome activation. Nat. Commun..

[74] Kalaiarasan P., Subbarao N., Bamezai R.N. (2014). Molecular simulation of Tyr105 phosphorylated pyruvate kinase M2 to understand its structure and dynamics. J. Mol. Model..

[75] Alquraishi M., Puckett D.L., Alani D.S., Humidat A.S., Frankel V.D., Donohoe D.R. (2019). Pyruvate kinase M2: a simple molecule with complex functions. Free Radic. Biol. Med..

[76] Anastasiou D., Yu Y., Israelsen W.J., Jiang J.K., Boxer M.B., Hong B.S. (2012). Pyruvate kinase M2 activators promote tetramer formation and suppress tumorigenesis. Nat. Chem. Biol..

