# Supplementary figures and images for: Epithelial redox stress programs macrophage immunometabolism through a ZNF24-MIF–NF–κB pathway in chronic nonbacterial prostatitis
Source: Redox Biol. 2026 Jan 20;90:104042. doi: 10.1016/j.redox.2026.104042 (PMC12859805; doi:10.1016/j.redox.2026.104042)

**A**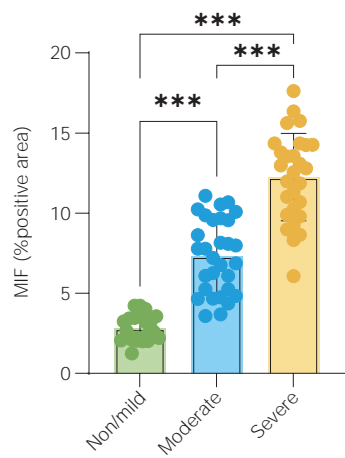**B**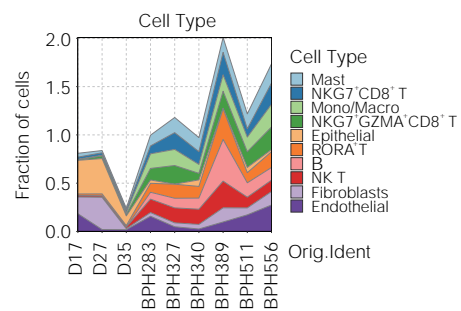**C**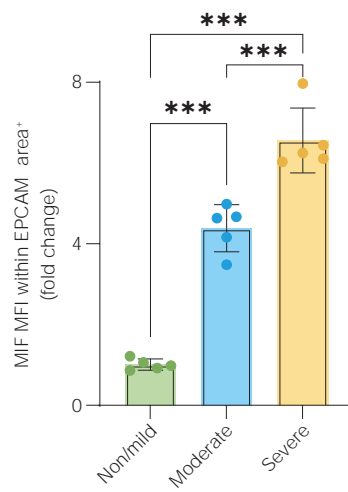**D**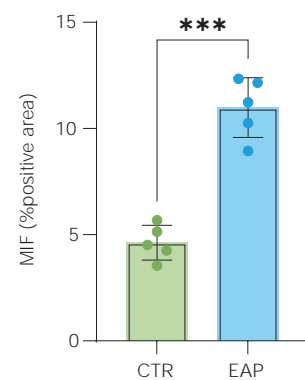**E**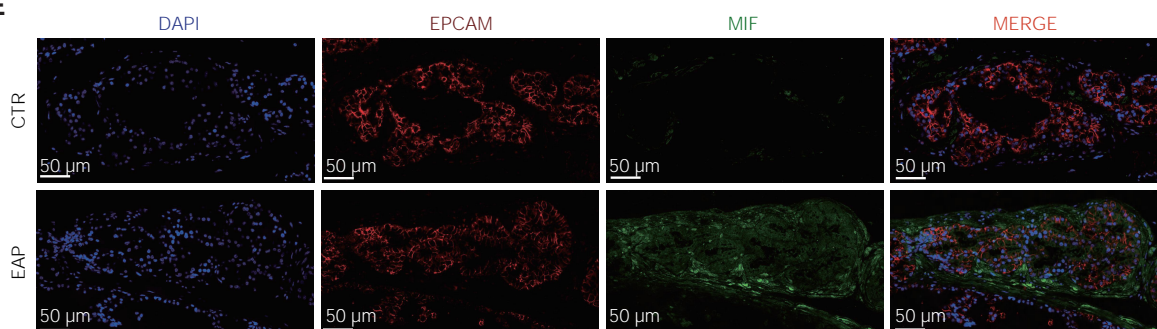**F**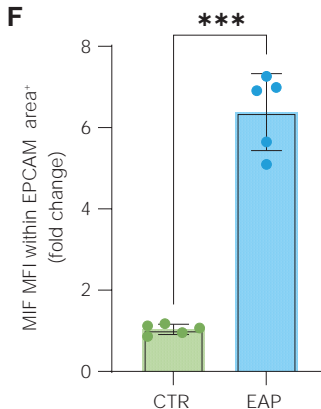

Supplement: Fig. S1 — Elevated epithelial MIF expression associates with prostate inflammation severity. (A) Quantification of MIF-positive areas in prostate tissues from BPH patients with mild, moderate, or severe inflammation. (B) Stacked area plot showing the relative proportions of major cell types across individual samples. (C) Quantification of MIF fluorescence intensity within the EPCAM+ epithelial compartment of BPH prostate tissues with increasing inflammation severity. (D) Quantification of MIF-positive areas in prostate tissues from control (CTR) and EAP mice. (E) Representative immunofluorescence (IF) images of mouse prostate sections showing co-localization of MIF (green) and EPCAM (red) in the prostatic epithelium of EAP mice. (F) Quantification of MIF fluorescence intensity within the EPCAM+ epithelial regions in prostate tissues from CTR and EAP mice. Data are presented as mean ± SD. ns, not significant; ∗p < 0.05; ∗∗p < 0.01; ∗∗∗p < 0.001. Abbreviations: CTR, control; EAP, experimental autoimmune prostatitis. [file mmc1.pdf]

nCount\_RNA

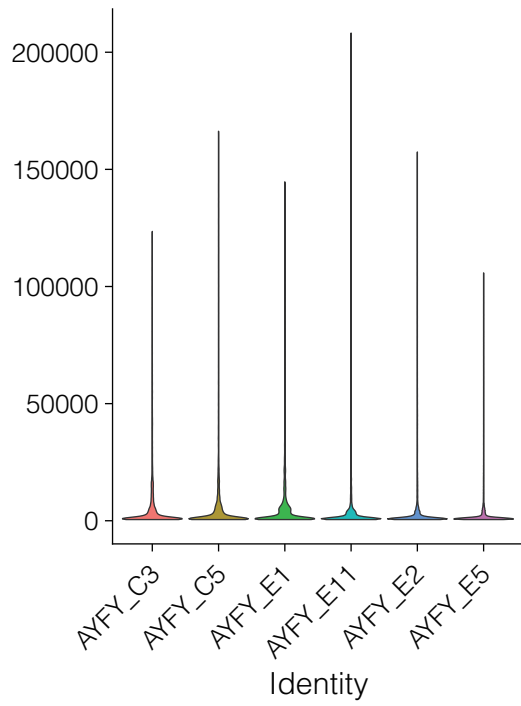

nFeature\_RNA

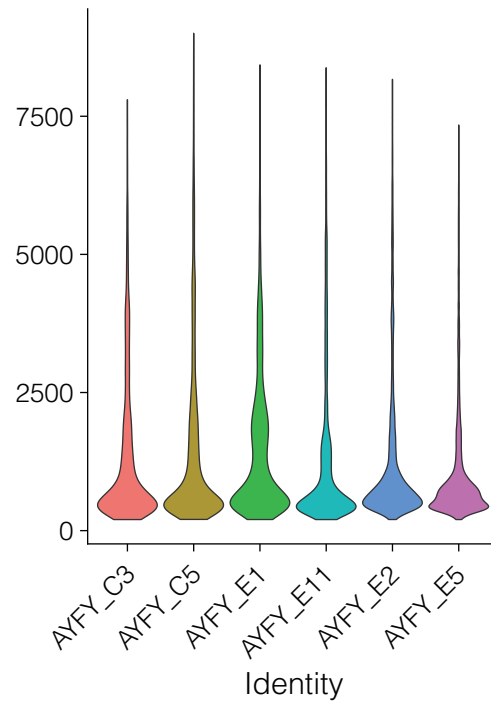

percent\_mito

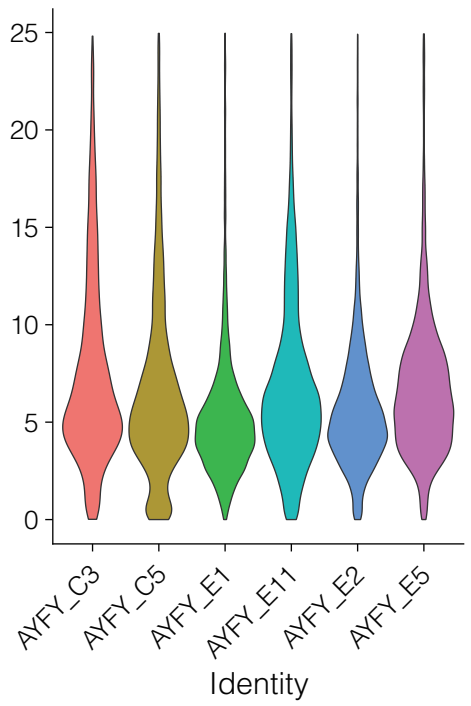

percent\_ribo

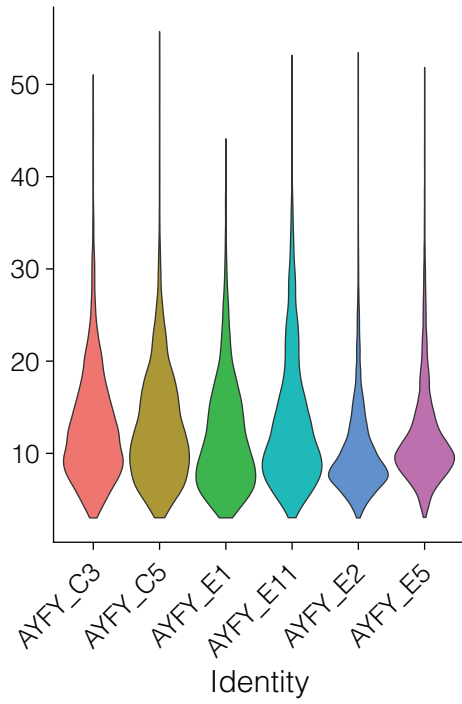

percent\_hb

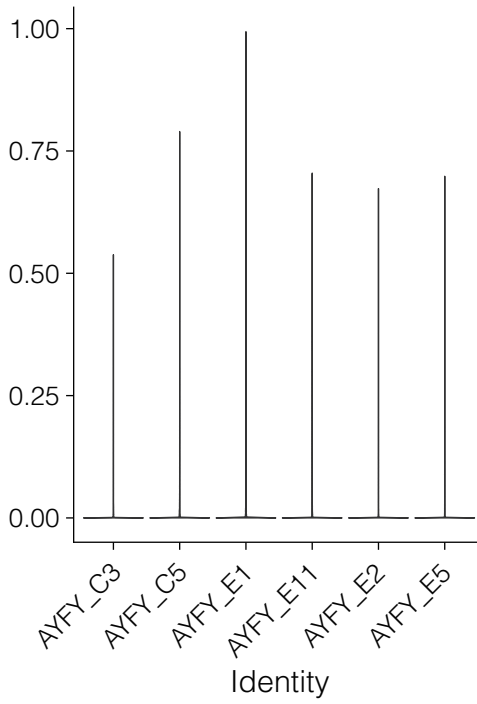

Supplement: Fig. S2 — Quality control metrics for the single-cell RNA-seq data. Violin plots displaying UMI counts, detected genes, and proportions of mitochondrial, ribosomal, and hemoglobin transcripts across six mouse prostate samples. [file mmc2.pdf]

A

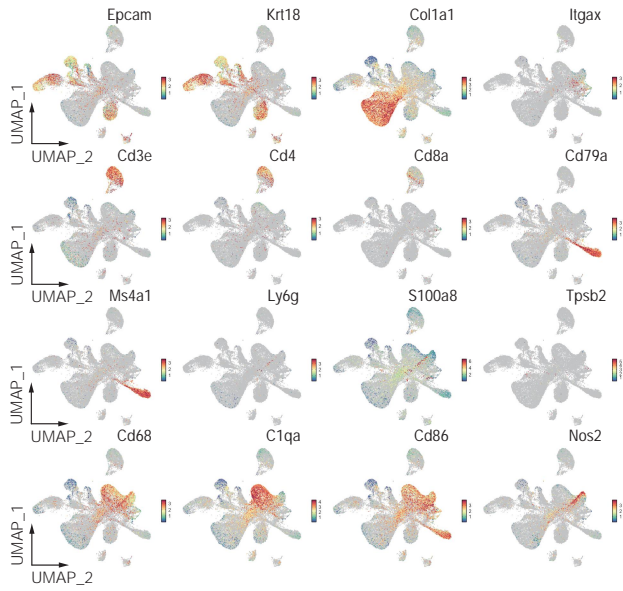

B

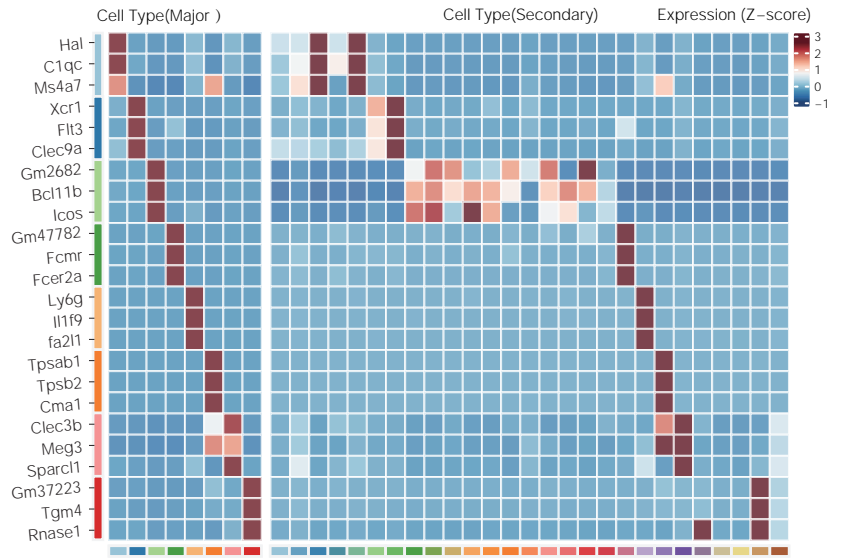

C

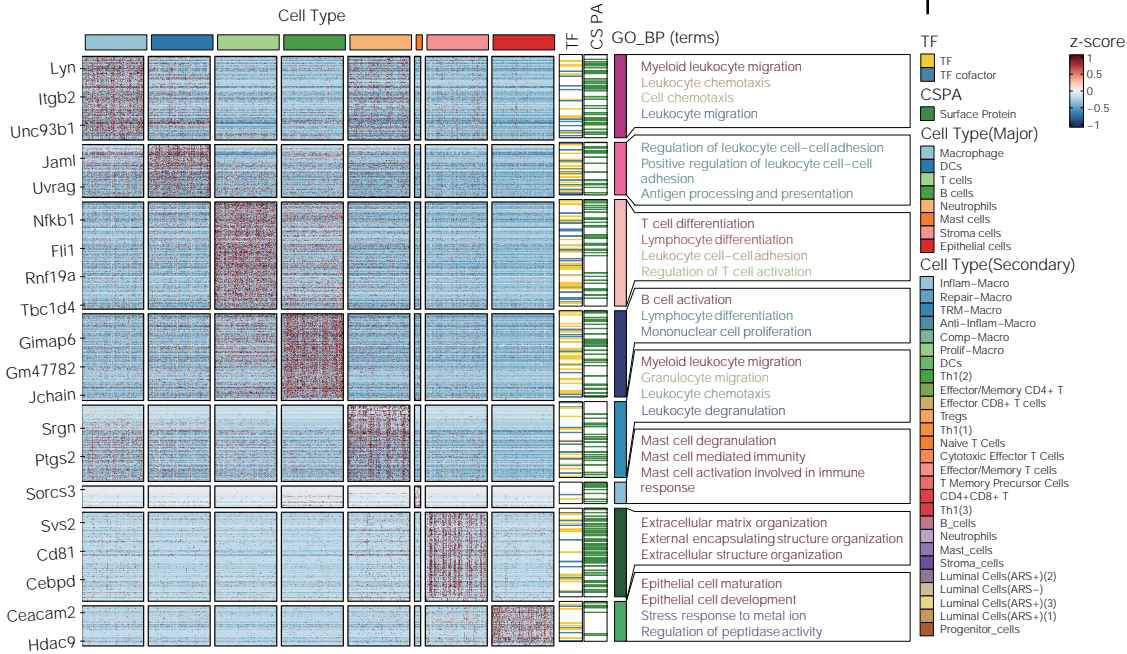

D

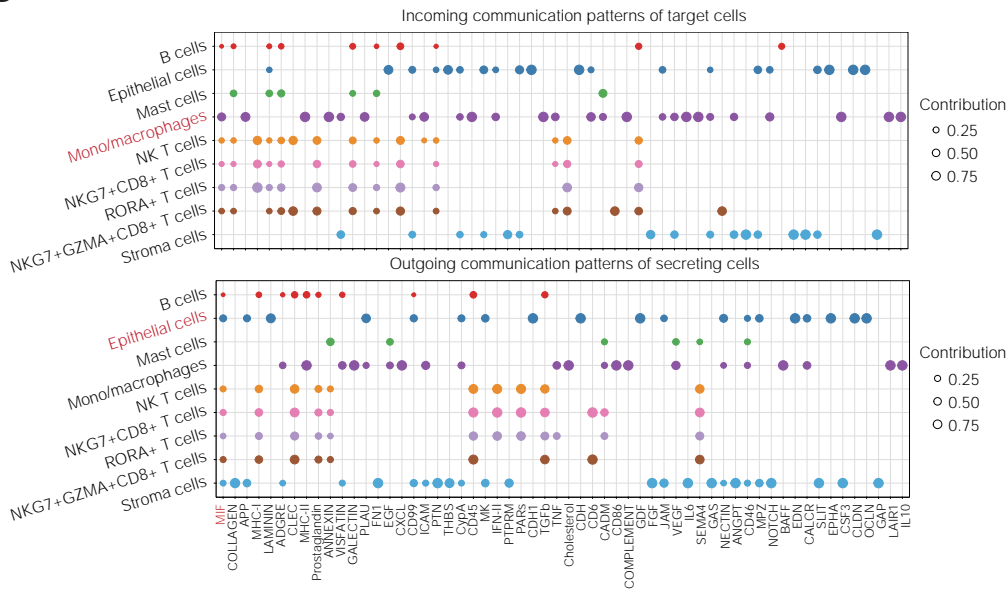

F

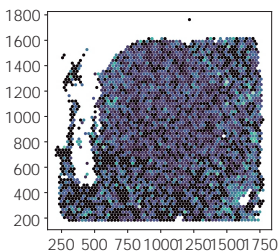

E

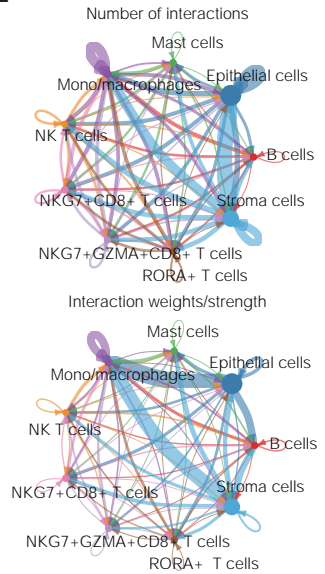

Supplement: Fig. S3 — Cell type annotation and functional characterization of mouse prostate single-cell RNA-seq data. (A) UMAP plots showing representative marker genes used to define major cell types, including epithelial (Epcam, Krt18), stromal (Col1a1), and immune populations (Cd3e, Cd79a, Itgax, Cd68), together with other lineage-specific markers. (B) Heatmap of marker gene expression (Z-score normalized) across major and secondary cell types, illustrating cell-type specificity and hierarchical relationships. (C) Functional enrichment analysis of cell type-specific genes, displaying representative genes, associated Gene Ontology biological process (GO_BP) terms, and functional annotations (e.g., immune activation, epithelial differentiation, and extracellular matrix remodeling). (D) Dot plots showing incoming (top) and outgoing (bottom) intercellular communication probabilities across major cell types, revealing prominent epithelial-to-macrophage signaling via MIF. (E) Network diagrams depicting the number (top) and strength (bottom) of inferred intercellular interactions among cell types. (F) Spatial distribution map showing Cell2location-inferred macrophage-associated spatial signals across prostate tissue sections. Regions enriched for MIF- and CD74-related expression patterns are indicated by increased signal intensity. Each spot represents estimates derived from spatial transcriptomics data. [file mmc3.pdf]

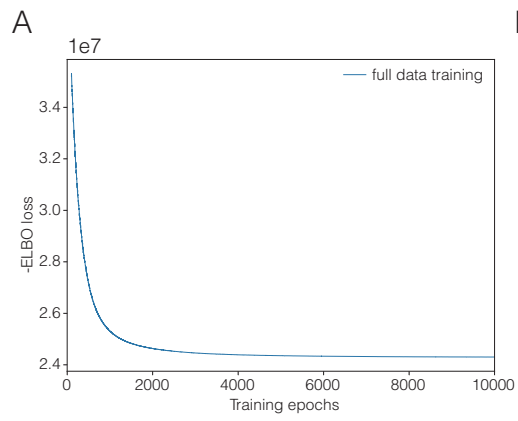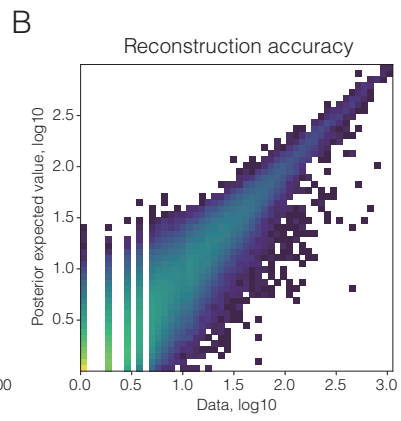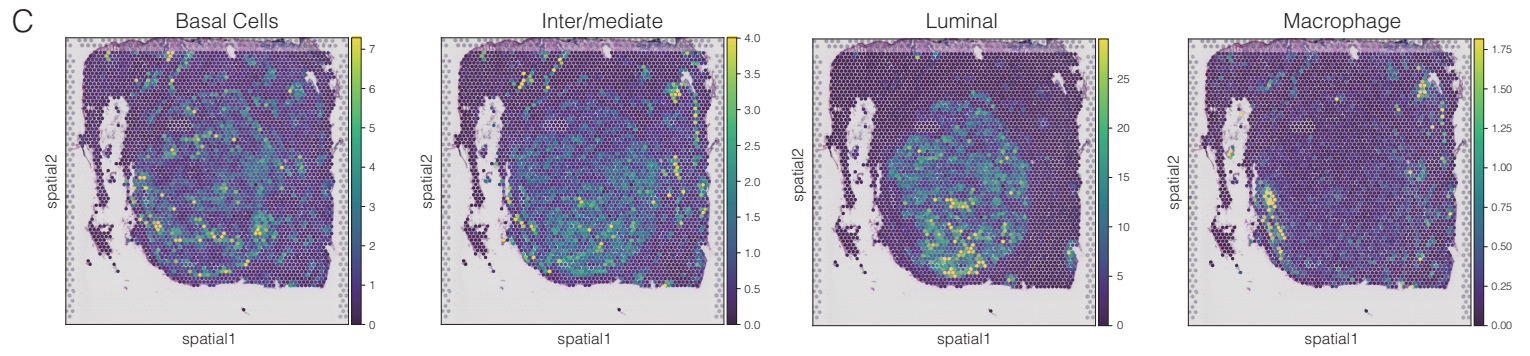

Supplement: Fig. S4 — Cell2location model training, reconstruction accuracy, and spatial mapping. (A) Training dynamics of cell2location showing the decline in negative evidence lower bound (-ELBO) loss across 10,000 epochs, indicating rapid optimization and convergence. (B) Reconstruction accuracy of the trained model, comparing posterior expected values with observed data (log10-transformed). The alignment along the diagonal reflects robust recovery of gene expression distributions. (C) Spatial distribution of representative epithelial (basal, intermediate, and luminal) and immune (macrophage) cell types inferred by cell2location, illustrating distinct tissue localization patterns. [file mmc4.pdf]

**A**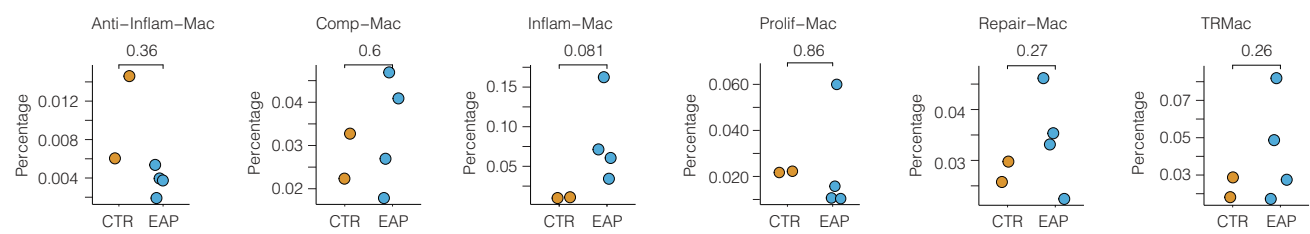**B**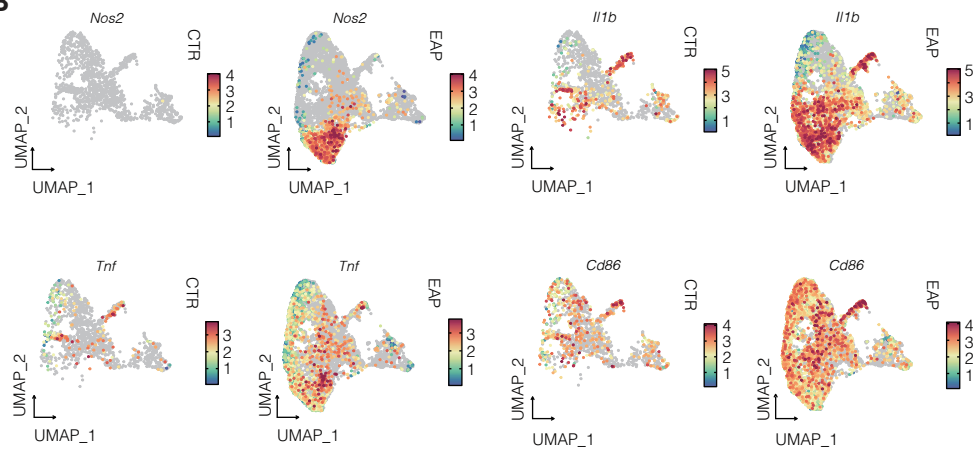

Supplement: Fig. S5 — Macrophage subpopulation distribution and M1-associated gene expression in CTR and EAP. (A) Frequencies of macrophage subpopulations in CTR and EAP. (B) UMAP feature plots of M1-associated genes (Nos2, Il1b, Tnf, Cd86) in CTR and EAP. Data are presented as the mean ± SD. ns, not significant. Abbreviations: CTR, control; EAP, experimental autoimmune prostatitis. [file mmc5.pdf]

**A**

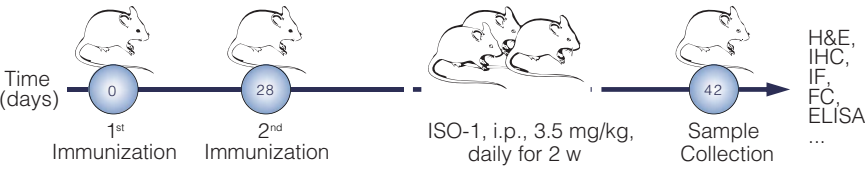

**B**

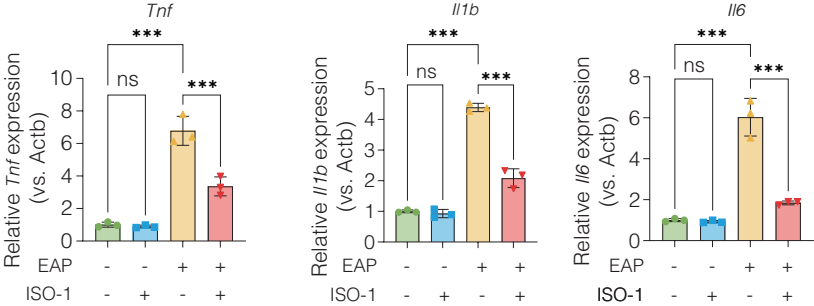

**C**

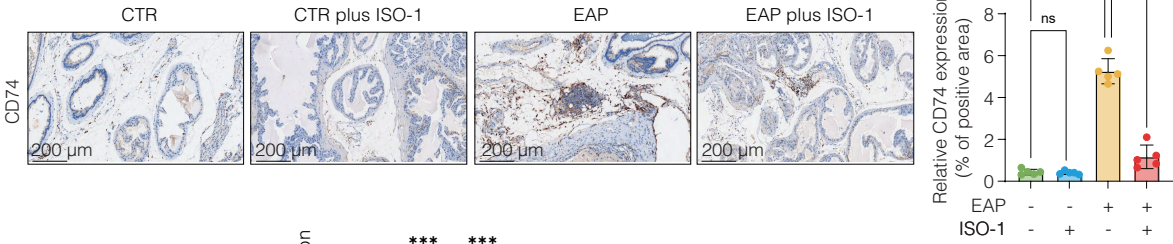

**D**

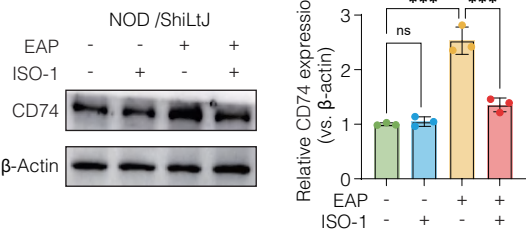

Supplement: Fig. S6 — ISO-1 treatment alleviates EAP-induced prostatic inflammation and downregulates CD74 expression in vivo. (A) Experimental timeline: CNP was induced by EAP (dual immunization), and ISO-1 (3.5 mg/kg, intraperitoneal injection) was administered daily for 2 weeks before tissue collection on day 42. (B) qRT‒PCR analysis showing suppression of Tnf, Il1b, and Il6 expression in prostate tissues. (C) IHC of CD74 in prostate tissues showing decreased expression with ISO-1. (D) Western blot analysis of CD74 protein in ISO-1-treated versus untreated EAP mice. Data are presented as the mean ± SD. ns, not significant; ∗p < 0.05; ∗∗p < 0.01; ∗∗∗p < 0.001. Abbreviations: CTR, control; EAP, experimental autoimmune prostatitis. [file mmc6.pdf]

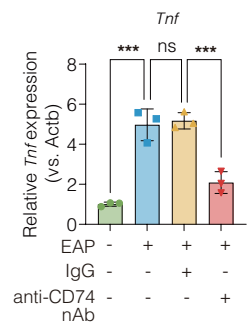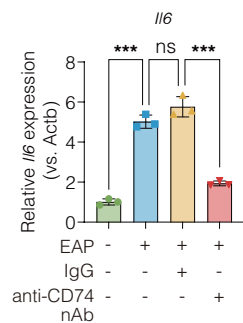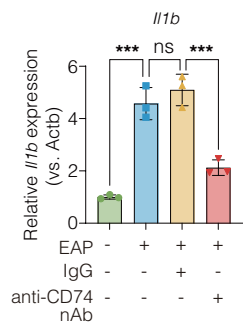

Supplement: Fig. S7 — Blockade of CD74 suppresses pro-inflammatory cytokine expression in EAP-induced prostate tissues. (A) qRT‒PCR analysis of Tnf, Il6, and Il1b expression in mouse prostate tissues, which was suppressed by the CD74 nAb. The data are presented as the means ± SD. ns, not significant; ∗p < 0.05; ∗∗p < 0.01; ∗∗∗p < 0.001. Abbreviations: CD74 nAb, anti-CD74 neutralizing antibodies; CTR, control; EAP, experimental autoimmune prostatitis; qRT‒PCR, quantitative real-time PCR. [file mmc7.pdf]

**A**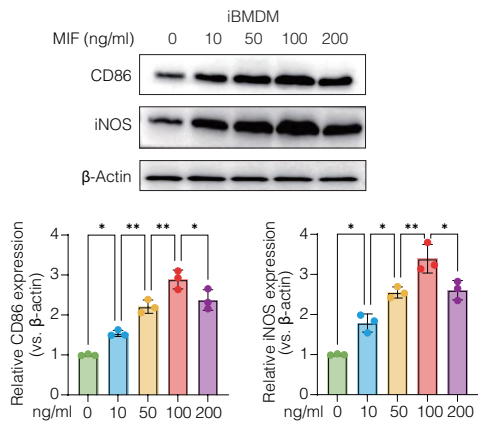**B**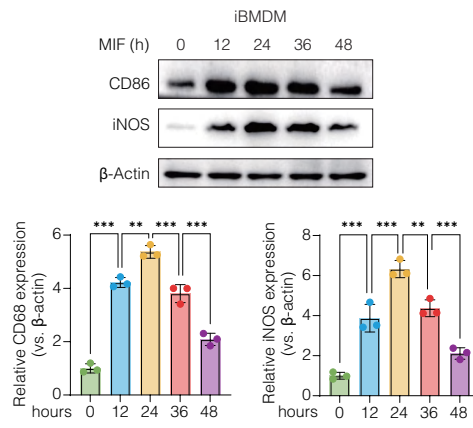**C**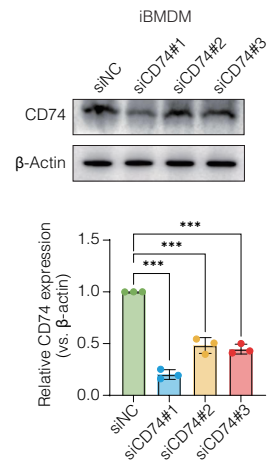**D**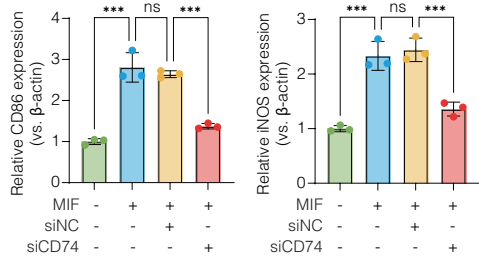**E**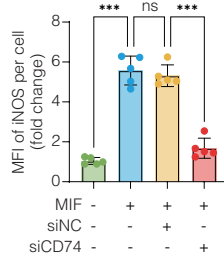**F**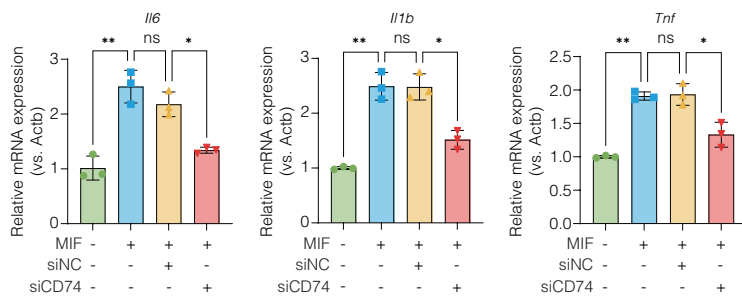**G**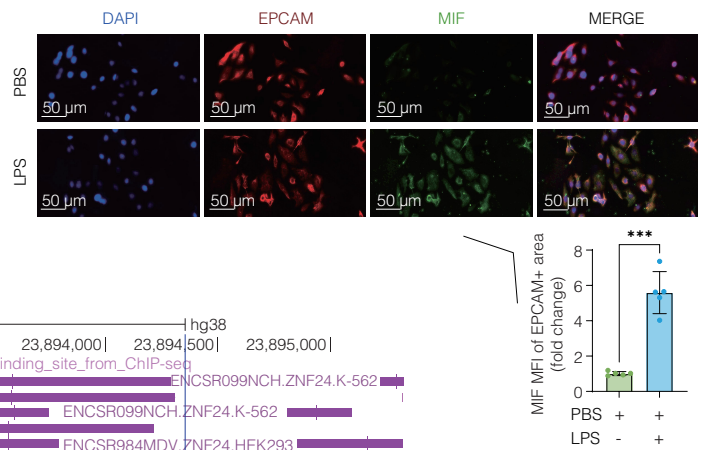**H**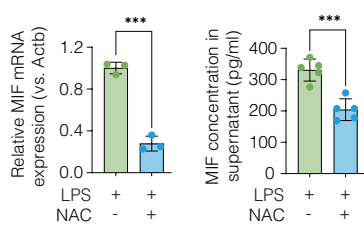**I**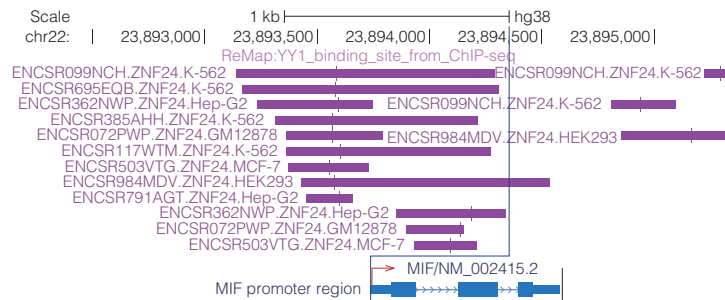**J**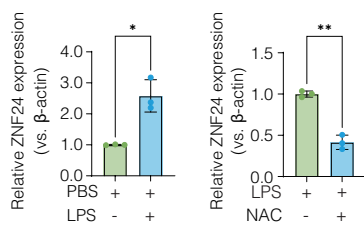**L**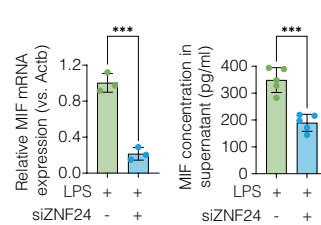**K**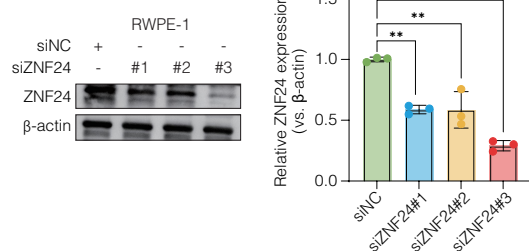

Supplement: Fig. S8 — An epithelial ROS–ZNF24–MIF axis promotes CD74-dependent macrophage M1 polarization. (A–B) Dose- and time-dependent induction of CD86 and iNOS in iBMDMs following MIF stimulation. (C–F) CD74 knockdown in iBMDMs suppresses MIF-induced M1 marker expression and proinflammatory cytokine production, as shown by western blot (C–D), iNOS immunofluorescence quantification (E), and qRT–PCR analysis of Il1b, Il6, and Tnf (F). (G) Immunofluorescence showing increased epithelial MIF expression in RWPE-1 cells after LPS stimulation. (H) ROS scavenging with NAC suppresses LPS-induced MIF expression and secretion in RWPE-1 cells, as assessed by qRT-PCR (left) and ELISA (right). (I) ZNF24 ChIP-seq peak enrichment at the MIF promoter based on ReMap 2022 datasets. (J–L) LPS-induced ZNF24 upregulation and ZNF24-dependent MIF expression in RWPE-1 cells, as shown by ZNF24 protein quantification (J), ZNF24 siRNA knockdown efficiency (K), and reduced MIF mRNA expression and secretion following ZNF24 silencing (L). Data are presented as mean ± SD. ns, not significant; ∗p < 0.05; ∗∗p < 0.01; ∗∗∗p < 0.001.Abbreviations: NAC, N-Acetylcysteine; ROS, Reactive Oxygen Species. [file mmc8.pdf]

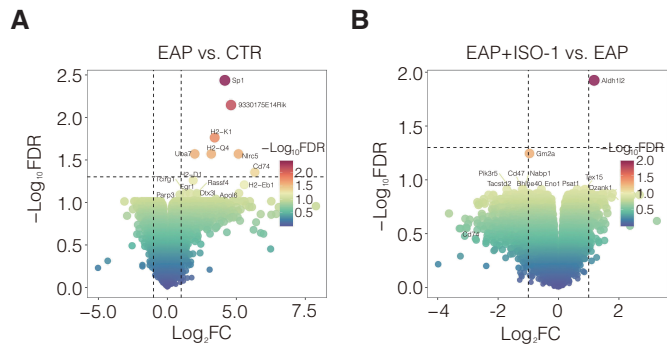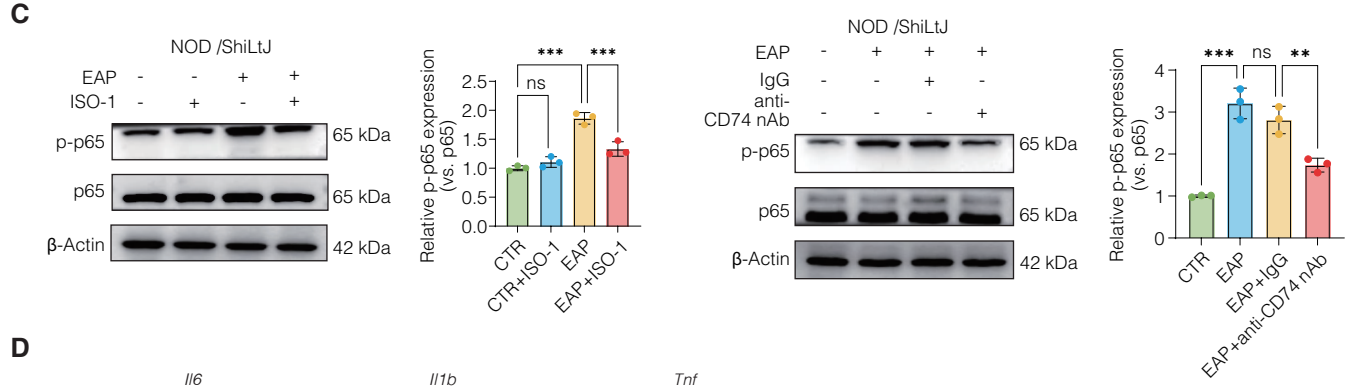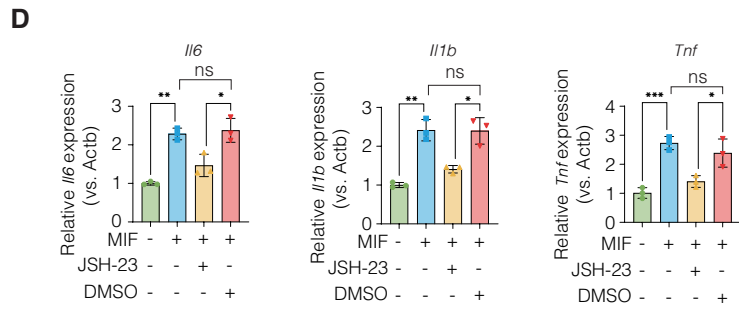

Supplement: Fig. S9 — Pharmacological inhibition of MIF or CD74 attenuates NF-κB activation and inflammatory signaling in EAP. (A-B) Volcano plots of differentially expressed genes in prostate tissues of EAP versus CTR (A) and ISO-1-treated versus untreated EAP (B). (C) Western blot of phosphorylated p65 (p-p65) in prostate tissues, elevated in EAP and reduced by ISO-1 or CD74 blockade. (D) qRT‒PCR showing that JSH-23 reverses MIF-induced Il6, Il1b, and Tnf transcription in iBMDMs. Data are presented as the mean ± SD. ns, not significant; ∗p < 0.05; ∗∗p < 0.01; ∗∗∗p < 0.001.Abbreviations: MIF, macrophage migration inhibitory factor; CTR, control; EAP, experimental autoimmune prostatitis; JSH-23, NF-κB inhibitor; DMSO, dimethyl sulfoxide. [file mmc9.pdf]

A

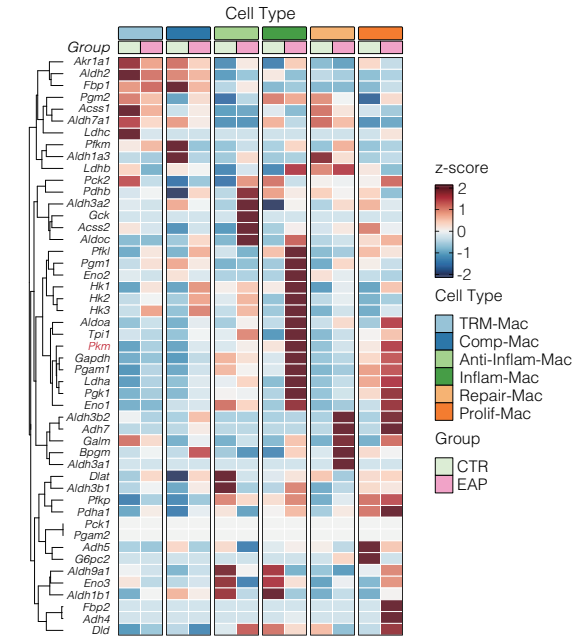

B

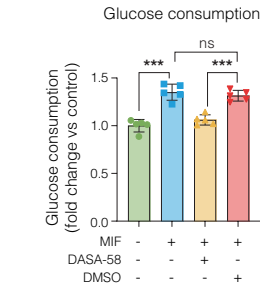

C

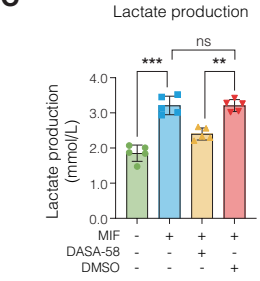

Supplement: Fig. S11 — MIF-CD74 signaling induces metabolic reprogramming toward glycolysis and impairs mitochondrial function in M1 macrophages. (A) Heatmap of glycolytic gene expression across cell types showing increased Pkm in inflammatory macrophages. (B–C) Glucose consumption and lactate production data showing that treatment with DASA-58 reversed MIF-induced glycolytic activation. The data are presented as the means ± SD. ns, not significant; ∗p < 0.05; ∗∗p < 0.01; ∗∗∗p < 0.001.Abbreviations: SD, standard deviation. [file mmc11.pdf]
