# Supplementary material for: Epithelial redox stress programs macrophage immunometabolism through a ZNF24-MIF–NF–κB pathway in chronic nonbacterial prostatitis
Source: Redox Biol. 2026 Jan 20;90:104042. doi: 10.1016/j.redox.2026.104042 (PMC12859805; doi:10.1016/j.redox.2026.104042)

A

Description	EDR confidence	Coverage	Score sequest HT	Expected q-value
Pkm2	High	19	20.29	< 0.0001
Myh9	High	6	10.66	< 0.0001
Rpl8	High	4	2.3	< 0.0001
Uba52	High	13	0	< 0.0001
Rps3a	High	6	0	< 0.0001

B

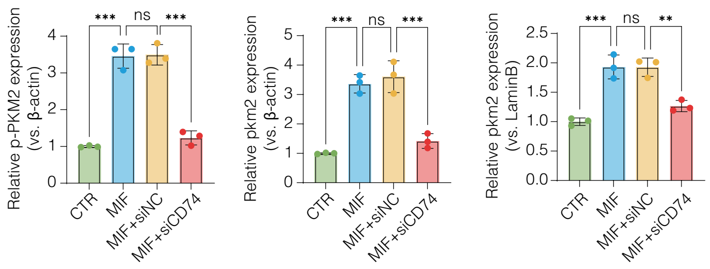

C

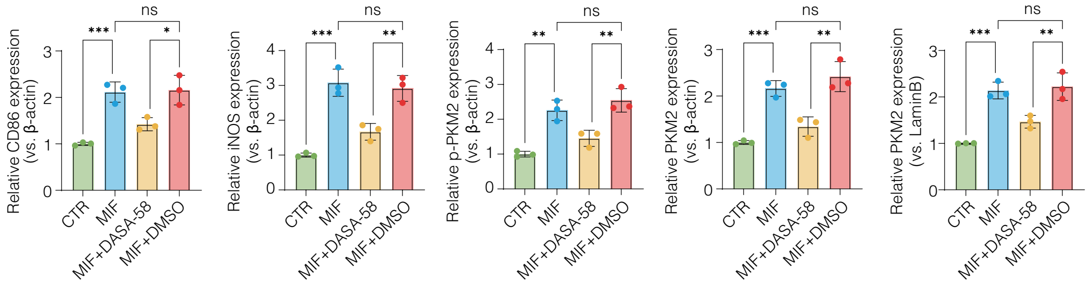

D

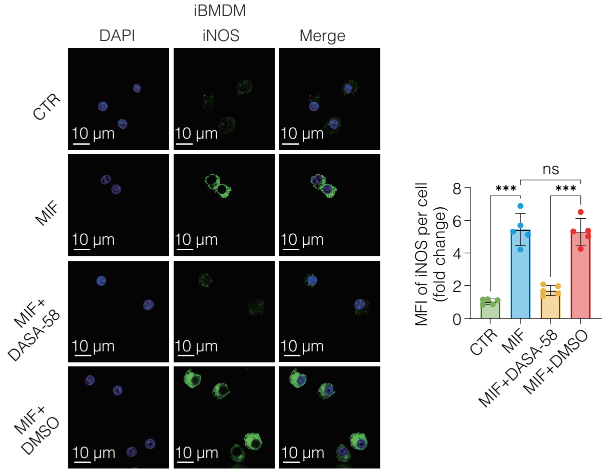

E

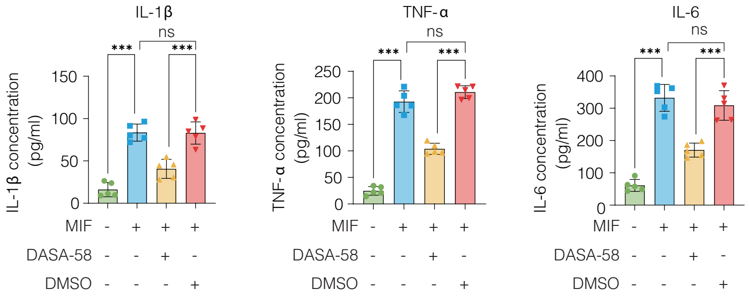

F

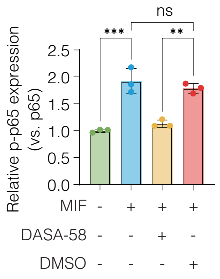

Supplement: Fig. S10 — MIF triggers NF-κB signaling through CD74-mediated PKM2 nuclear import. (A) Table of high-confidence CD74-binding candidates from mass spectrometry, including PKM2. (B) Quantification of PKM2 and p- PKM2 protein levels in iBMDMs. (C) Quantification of CD86, iNOS, PKM2 and p- PKM2 protein levels in iBMDMs. (D) Immunofluorescence showing that DASA-58 blocks MIF-induced iNOS expression. (E) ELISA showing the suppression of MIF-induced IL-1β, IL-6, and TNF-α secretion by DASA-58. (F) Quantification of p-p65 protein levels in iBMDMs. The data are presented as the means ± SD. ns, not significant; ∗p < 0.05; ∗∗p < 0.01; ∗∗∗p < 0.001.Abbreviations: DASA-58, PKM2 tetramer stabilizer; CHX, cycloheximide; SD, standard deviation; DMSO, dimethyl sulfoxide. [file mmc10.pdf]
