# Supplementary material for: Epithelial redox stress programs macrophage immunometabolism through a ZNF24-MIF–NF–κB pathway in chronic nonbacterial prostatitis
Source: Redox Biol. 2026 Jan 20;90:104042. doi: 10.1016/j.redox.2026.104042 (PMC12859805; doi:10.1016/j.redox.2026.104042)
Supplement: Multimedia component 12 [file mmc12.docx]

| **Table S1. Criteria for grading inflammation in prostate tissues from patients with benign prostatic hyperplasia.** | |
| --- | --- |
| **Grading criteria** | **Morphology description (typical inflammatory cell density, cells/mm^2^)** |
| 1/Mild | Single inflammatory cells, most separated by distinct spaces (< 100). |
| 2/Moderate | Confluent patchy inflammatory cells without tissue destruction or lymphoid nodules/follicle formation (100-500). |
| 3/Severe | Inflammatory cell clusters with tissue destruction or lymphoid nodules/follicle formation (> 500). |
