# Supplementary material for: Epithelial redox stress programs macrophage immunometabolism through a ZNF24-MIF–NF–κB pathway in chronic nonbacterial prostatitis
Source: Redox Biol. 2026 Jan 20;90:104042. doi: 10.1016/j.redox.2026.104042 (PMC12859805; doi:10.1016/j.redox.2026.104042)
Supplement: Multimedia component 13 [file mmc13.docx]

**Table S2. Criteria for grading prostatic inflammation in Experimental Autoimmune Prostatitis mouse tissues.**

| **Degree(score)** | **Description** |
| --- | --- |
| 0 | no inflammation |
| 1 | Mild vascular hyperplasia with clear mononuclear cell infiltration in the interstitium |
| 2 | Moderate vascular hyperplasia accompanied by mononuclear cell infiltration in the interstitium |
| 3 | Severe vascular hyperplasia, hemorrhage, and widespread mononuclear cell infiltration in the interstitium |
