# Supplementary material for: Epithelial redox stress programs macrophage immunometabolism through a ZNF24-MIF–NF–κB pathway in chronic nonbacterial prostatitis
Source: Redox Biol. 2026 Jan 20;90:104042. doi: 10.1016/j.redox.2026.104042 (PMC12859805; doi:10.1016/j.redox.2026.104042)
Supplement: Multimedia component 14 [file mmc14.docx]

**Table S3. Primers and siRNA sequences used in this study.**

| **Gene** | **Forward** | **Reverse** | | |
| --- | --- | --- | --- | --- |
| **Human primers** | | |  |  |
| *MIF* | CCGGACAGGGTCTACATCAA | TTAGGCGAAGGTGGAGTTGT | | |
| *ACTB* | CCTGGCACCCAGCACAAT | GGGCCGGACTCGTCATAC | | |
| **Mouse primers** | | |  |  |
| *Il6* | CTTCTTGGGACTGATGCTGGTGAC | TCTGTTGGGAGTGGTATCCTCTGTG | | |
| *Tnf* | CGCTCTTCTGTCTACTGAACTTCGG | GTGGTTTGTGAGTGTGAGGGTCTG | | |
| *Il1b* | CACTACAGGCTCCGAGATGAACAAC | TGTCGTTGCTTGGTTCTCCTTGTAC | | |
| *Actb* | GGCTGTATTCCCCTCCATCG | CCAGTTGGTAACAATGCCATGT | | |
| **Mouse siRNA sequence** | | | |  |
| si*Cd74*-1 | 5′-CGACGAGAACGGUAACUAUTT-3′ | | | |
| si*Cd74*-2 | 5′-CACCUAAAGUACUGACCAATT-3′ | | | |
| si*Cd74*-3 | 5′-CGUCCAAUGUCCAUGGAUATT-3′ | | | |
| siNC | 5′-UUCUCCGAACGUGUCACGUTT-3′ | | | |
| **Human siRNA sequence** | | | | |
| si*ZNF24-1* | 5′-CUCGAAAGAAACAACAUAUAUTT-3′ | | | |
| si*ZNF24-2* | 5′-UCAGUGGAAGAAGAUUCAAUATT-3′ | | | |
| si*ZNF24-3* | 5′-UCGUAUAGUCAAAGCUCAAAUTT-3′ | | | |
| siNC | 5′-UUCUCCGAACGUGUCACGUTT-3′ | | | |
