# Supplementary material for: Epithelial redox stress programs macrophage immunometabolism through a ZNF24-MIF–NF–κB pathway in chronic nonbacterial prostatitis
Source: Redox Biol. 2026 Jan 20;90:104042. doi: 10.1016/j.redox.2026.104042 (PMC12859805; doi:10.1016/j.redox.2026.104042)
Supplement: Multimedia component 15 [file mmc15.docx]

**Table S4. Antibodies used for immunofluorescence, immunoblotting, and flow cytometry.**

| **Antibodies** | **Company** | **Catalog Number** |
| --- | --- | --- |
| MIF | Abcam | Cat#ab227073 |
| EPCAM | Abcam | Cat#ab213500 |
| CD74 | Abcam | Cat#ab289885 |
| CD74 | Abcam | Cat#ab108393 |
| CD86 | Affinity | Cat#DF6332 |
| CD45 | Affinity | Cat#DF6839 |
| CD68 | Abcam | Cat#ab303565 |
| iNOS | Affinity | Cat#AF0199 |
| ZNF24 | Proteintech | Cat#11219-1-AP |
| 8-OHDG | Servicebio | Cat#GB150115 |
| Rabbit anti-mouse CD11b-APC | BioLegend | Cat#101212 |
| Rabbit anti-mouse CD86-PE | BioLegend | Cat#105008 |
| Rabbit anti-mouse F4/80-APC | BioLegend | Cat#123108 |
| β-Actin | Cell Signaling Technology | Cat#4967 |
| Phospho-NF-κB p65 | Affinity | Cat#AF2006 |
| NF-κB p65 | Cell Signaling Technology | Cat#8242 |
| PKM2 | Proteintech | Cat#60268-1-Ig |
| UB | Proteintech | Cat#10201-2-AP |
| Phospho-PKM2 | Cell Signaling Technology | Cat#3827 |
| PKM2 | Proteintech | Cat#15822-1-AP |
| CD74 | Raybiotech | Cat#188-10050-1 |
| Lamin B | Abcam | Cat#ab16048 |
| Goat Anti-Mouse IgG  (H+L)-HRP Conjugate | Bio-Rad | Cat#1721011 |
| Goat Anti-Rabbit IgG (H+ L)-HRP Conjugate | Bio-Rad | Cat#1706515 |
| HRP-conjugated goat anti-rabbit | Servicebio | Cat#GB23303 |
| Normal mouse IgG | Millipore sigma | Cat#12-31 |
| cy3 goat anti-rabbit antibody | Servicebio | Cat#GB21303 |
| alexa Fluor 488 goat anti-rabbit antibody | Servicebio | Cat#GB25303 |
| Normal Rabbit IgG | Cell Signaling Technology | Cat#2729 |
