# Supplementary material for: Epithelial redox stress programs macrophage immunometabolism through a ZNF24-MIF–NF–κB pathway in chronic nonbacterial prostatitis
Source: Redox Biol. 2026 Jan 20;90:104042. doi: 10.1016/j.redox.2026.104042 (PMC12859805; doi:10.1016/j.redox.2026.104042)
Supplement: Multimedia component 16 [file mmc16.docx]

**Table S5. Reagents and kits used in this study.**

| **Product name** | **Company** | **Catalog Number** |
| --- | --- | --- |
| Complete Freund’s adjuvant | Sigma‒Aldrich | Cat#F5881 |
| Recombinant mouse MIF | CHAMOT | Cat#CM059-20MP |
| ISO-1 | MedChemExpress | Cat#HY-16692 |
| Anti-CD74-neutralizing antibodies | BD Pharmingen | Cat#555317 |
| DMEM | Thermo Fisher Scientific | Cat#10564011 |
| Fetal bovine serum | Thermo Fisher Scientific | Cat#26140079 |
| Penicillin‒streptomycin | Thermo Fisher Scientific | Cat#15140122 |
| RIPA buffer | Sigma‒Aldrich | Cat#R0278 |
| Protease and phosphatase inhibitor cocktail | Thermo Fisher Scientific | Cat#1861281 |
| JSH-23 | MedChemExpress | Cat#HY-13982 |
| NAC | MedChemExpress | Cat#HY-B0215 |
| DASA-58 | MedChemExpress | Cat#HY-19330 |
| Lipopolysaccharides | MedChemExpress | Cat#HY-D1056 |
| KM medium | ScienCell | Cat#2101 |
| Pierce ECL substrate | Thermo Fisher Scientific | Cat#21050 |
| DAB chromogenic kit | Solarbio | Cat#DA1016 |
| PrimeScript^TM^ RT reagent Kit | Takara | Cat#RR047A |
| TB Green® Premix Ex Taq^TM^ kit | Takara | Cat#RR820A |
| TNF-α ELISA kit | Elabscience | Cat#E-EL-M3063 |
| IL-1β ELISA kit | Elabscience | Cat#E-EL-M0037 |
| IL-6 ELISA kit | Elabscience | Cat#E-EL-M0044 |
| MIF ELISA kit | Elabscience | Cat# E-EL-M0771 |
| MIF ELISA kit | Elabscience | Cat# E-EL-H6170 |
| Six-well co-culture plate | Corning | Cat#3412 |
| DMEM/F-12 | Gibco | Cat#11330032 |
| JC-1 Assay Kit | Beyotime | Cat#C2006 |
| Lactate detection kit | Nanjing jiancheng | Cat#A019-2-1 |
| Glucose consumption detection kit | Nanjing jiancheng | Cat#F006-1-1 |
| Immunoprecipitation Kit with Protein A/G Magnetic Beads | Thermo Fisher Scientific | Cat#88804 |
| Coomassie Brilliant Blue | Thermo Fisher Scientific | Cat#20279 |
| Nuclear Extract Kit | Beyotime | Cat#P0027 |
| SimpleChIP® Enzymatic Chromatin IP Kit | Cell Signaling Technology | Cat#9003 |
| ROS detection kit | Beyotime | Cat#S0033S |
