# Supplementary material for: Epithelial redox stress programs macrophage immunometabolism through a ZNF24-MIF–NF–κB pathway in chronic nonbacterial prostatitis
Source: Redox Biol. 2026 Jan 20;90:104042. doi: 10.1016/j.redox.2026.104042 (PMC12859805; doi:10.1016/j.redox.2026.104042)
Supplement: Multimedia component 17 [file mmc17.docx]

**Table S6. Baseline characteristics of healthy volunteers and patients with chronic prostatitis–like symptoms.**

| **Variable** | **Overall (n=53)** | **Healthy control (n=13)** | **CP-LS (n=40)** | ***P* value** |
| --- | --- | --- | --- | --- |
| Age, median, mean (+SD) | 29.47 ± 3.98 | 30.00 ± 4.34 | 29.30 ± 3.90 | 0.587 |
| BMl, median, mean (+SD) | 24.27 ± 2.27 | 23.84 ± 2.28 | 24.40 ± 2.28 | 0.443 |
| Pain score, median (IQR) | 11 (4-14.5) | 0 (0-0) | 12 (10-15) | <0.001 |
| NIH-CPSl Score, median (IQR) | 25 (6.5-29.5) | 0 (0-0) | 27 (21.25-30.75) | <0.001 |
| Course, m, median (IQR) | 12 (1.5-44) | 0 (0-0) | 24 (8.75-45) | <0.001 |

**Note:** CP-LS, chronic prostatitis-like symptoms; IQR, interquartile range; BMI, body mass index; NlH-CPSl, National lnstitutes of Health Chronic Prostatitis Symptom index; SD, standard deviation.
